# Hardware implementation of backpropagation using progressive gradient descent for in situ training of multilayer neural networks

**DOI:** 10.1126/sciadv.ado8999

**Published:** 2024-07-12

**Authors:** Eveline R. W. van Doremaele, Tim Stevens, Stijn Ringeling, Simone Spolaor, Marco Fattori, Yoeri van de Burgt

**Affiliations:** ^1^ Department of Mechanical Engineering and Institute for Complex Molecular Systems, Eindhoven University of Technology, Eindhoven 5612AP, Netherlands.; ^2^ Eindhoven Hendrik Casimir Institute, Eindhoven University of Technology, Eindhoven 5612AP, Netherlands.; ^3^ Department of Electrical Engineering, Eindhoven University of Technology, Eindhoven 5612AP, Netherlands.

## Abstract

Neural network training can be slow and energy-expensive due to the frequent transfer of weight data between digital memory and processing units. Neuromorphic systems can accelerate neural networks by performing multiply-accumulate operations in parallel using nonvolatile analog memory. However, executing the widely used backpropagation training algorithm in multilayer neural networks requires information—and therefore storage—of the partial derivatives of the weight values preventing suitable and scalable implementation in hardware. Here, we propose a hardware implementation of the backpropagation algorithm that progressively updates each layer using in situ stochastic gradient descent, avoiding this storage requirement. We experimentally demonstrate the in situ error calculation and the proposed progressive backpropagation method in a multilayer hardware-implemented neural network. We confirm identical learning characteristics and classification performance compared to conventional backpropagation in software. We show that our approach can be scaled to large and deep neural networks, enabling highly efficient training of advanced artificial intelligence computing systems.

## Teaser

Backpropagation with progressive gradient descent enables fast and efficient training in hardware neural networks.

## INTRODUCTION

Neural networks have become increasingly popular due to their ability to solve complex problems by processing and structuring large amounts of data. However, their growing size and complexity have resulted in an exponential increase in the computational power (and energy) required to process the information. This has led to the limitations of traditional computing architectures, where the processing and memory units are separated. To address these limitations, neuromorphic computing has emerged as a promising alternative. This brain-inspired architecture allows for efficient vector-matrix multiplications and has been successful in accelerating neural networks using in-memory computing (*1*–*4*). The weights of the neural networks are represented by the conductance of neuromorphic devices or memristors, which have been demonstrated in a wide variety of materials and systems, ranging from phase change memory (*4*), resistive random access memory (*1*, *5*), electrochemical random access memory (EC-RAM) (*6*–*9*), etc. While neuromorphic systems can accelerate computing through parallel operations in nonvolatile analog memory, training these systems still faces challenges in hardware implementation. Commonly, the neural network is trained in software ex situ, and subsequently the weights are transferred directly to the neuromorphic devices by mapping conductance values. To achieve an accuracy comparable to software neural networks, the weights need to be precisely programmed with the correct conductance values. This can be challenging due to the stochastic tuning behavior of common memristors, resulting in the need for sophisticated, high-resolution, and relatively slow, closed-loop tuning mechanisms with sequential programming, which eliminates the efficiency gained by hardware-based systems. Once trained, these circuits display a high retention and long-term inference, but for transfer, edge, and online learning systems, it is essential that devices can be accurately and rapidly tuned. Various other researches have been reported on improved programming schemes to increase the programming efficiency and accuracy (*4*, *10*–*14*). However, hardware imperfections, such as defective devices and parasitic wire resistance and capacitance, are inevitable and are the main cause of inferior hardware neural network performance. Furthermore, ex situ training also restricts the learning capabilities to software-based systems. In situ training, on the other hand, can adapt the weights and compensate for these imperfections automatically (*15*, *16*) and allows for faster and more scalable computing systems (*17*).

Alternatively, hybrid training methods have also been used to adapt to device imperfections and improve the overall system performance. After the ex situ trained weights are transferred to the neuromorphic devices, the weights of one (or more) layers are reprogrammed using the backpropagated gradients calculated from hardware-measured outputs (*18*). Another hybrid method uses the hardware measured outputs for training in software to progressively program the weights layer-by-layer (*19*). While these strategies are efficient in mitigating accuracy loss due to hardware imperfections, they still require computationally expensive ex situ training by calculating the weight update and programming the update using software. In an effort to allow in situ training in hardware motivated by the efficient learning process of the brain, a sequential forward-forward algorithm has recently been proposed (*20*). This algorithm has several advantages that allow for a more straightforward hardware implementation; however, its simulated performance is insufficient to replace the established backpropagation algorithm. Methods to perform in situ backpropagation have been proposed but are often limited to single layers (*21*, *22*) or require a binary update step (*23*, *24*) or binary weights (*25*, *26*). Backpropagation using (stochastic) gradient descent allows one to differentiate between the weights and update them in proportion to their relative contribution to the error, ensuring maximum error reduction. Compared to update mechanisms with the same magnitude, gradient descent allows for more efficient weight updates and an overall better neural network (classification) performance. On the other hand, gradient descent is more complex to execute in hardware due to the required calculation of partial derivatives and digital storage of local error gradients and weight values (*27*) (for multilayer neural networks) (*4*, *14*). Here, we propose an approach to integrate backpropagation directly on the chip using hardware-based stochastic gradient descent suitable for multilayer neural networks. We introduce a method to calculate the partial derivatives of the weights locally, which allows for efficient on-chip weight updates during the training (backpropagation) phase. Our proposed method enables autonomous learning capabilities for efficient and straightforward implementation of transfer, edge, and online learning systems directly in hardware.

## RESULTS

### Hardware implementation gradient descent

**Fig. 1.**
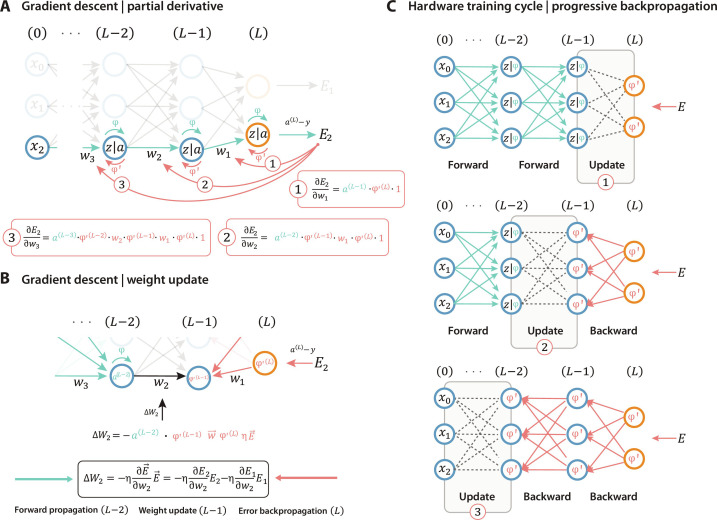
Gradient descent for progressive backpropagation. (**A**) Neural network architecture demonstrating the partial derivative of the error with respect to various weights. (**B**) Calculation of the weight update that is equal to the product of the forward and backward signal. (**C**) Approach to realize the forward and backward signal multiplication by progressively updating the weights of each layer.

The proposed mechanism works by calculating the weight change layer-by-layer and update this directly in hardware. We use the feedforward and error signal passing through each layer sequentially while updating each layer immediately, avoiding the need to store any additional parameters. This approach differs fundamentally from previous reports using outer product weight updates, in which the local error gradients (and intermediate neuron activation) are stored digitally (*4*, *28*). During feedforward (inference), the output *z* of layer *L* is calculated through a vector-matrix multiplication of the input times the weights (*w^L^*) and an activation function *a^L^* = φ(*z^L^*), with the input equal to *a*^*L*−1^. The weight update for each weight is relative to its contribution to the error *E* and calculated according to $\text{∆}w_{\mathrm{ij}}^{L}=-\eta \frac{\partial \vec{E}\;}{\partial w_{\mathrm{ij}}^{L}}\vec{E}$, where η is the learning rate, a hyperparameter used for scaling the update step. The error is determined by a selected loss function, which, in this case, equals the output (*a^L^*) minus the target (*y*). For a single-layer neural network (e.g., perceptron), the update is relatively straightforward since this only depends on the input and output, which are directly available in hardware. Only the derivative of the activation function (φ′) needs to be calculated, which is either 1 or 0 [in the case of a rectified linear unit (ReLU)] (see $\frac{\partial E_{2}}{\partial w_{1}}$ in Fig. 1A).

**Fig. 2.**
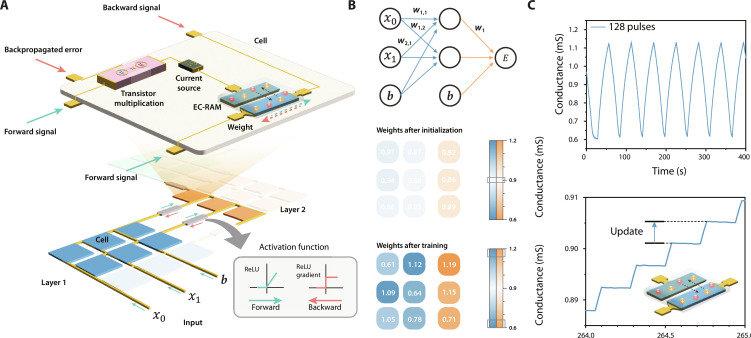
Hardware neural network. (**A**) Illustration of multiple crossbars including the cells with the neuromorphic EC-RAM devices, transistor multiplication, and current source and ReLU activation function, representing a hardware neural network. (**B**) Schematic of the 2 × 2 × 1 neural network with the conductance values of the weights before and after training indicated. Blue and orange represent the weights of layers 1 and 2, respectively. (**C**) Conductance modulation of the EC-RAM regulated using a current source.

However, in a multilayer neural network, the weight update is influenced by the surrounding layers and depends on the activation of the previous neurons (*a*^*L*−2^) and the weights in posterior layers (*w^L^*) for weights in layer *L* − 1. As a result, the gradient descent algorithm typically requires (digital) storing of the neuron activation (intermediate outputs) and local error gradients, next to the weight values, to apply an outer product weight update step (*4*, *29*), which is undesirable and unpractical for hardware neural networks. The implementation method we propose here allows one to circumvent this restriction by progressively updating a single layer (see Fig. 1C). We calculate the weight update by applying the feedforward and the backpropagation signal at the same time (see Fig. 1B). The feedforward signal travels downstream through the network (representing *a*^*L*−2^) until the layer (*L* − 1) that requires updating. The error propagates backward (upstream) through the network until the layer *L* − 1 and is therefore multiplied by the weights ( ${\vec{w}}^{L}$ ) (see also Supplementary Text 1). To experimentally demonstrate and validate this progressive approach in multilayer neural networks, we developed and fabricated a two-layer neural network based on organic EC-RAM (*6*) (see Fig. 2). While the algorithm is universal across various kinds of memory devices, the three-terminal EC-RAM displays excellent and predictable characteristics (Fig. 2C) that are essential for in situ training (*28*). These characteristics include linear and symmetric conductance tuning (*30*), with low write noise, and have been demonstrated to allow parallel updating (*13*) and be CMOS (complementary metal-oxide semiconductor) compatible (*9*). To facilitate the multiplication, we use a transistor operated in triode region, where the drain current is linearly proportional to the gate voltage (feedforward signal) and the drain voltage (backflowing error). The transistor, located at every weight, allows for efficient analog vector-vector multiplications and enables parallel weight updates for each layer (see Supplementary Text 1).

### In situ hardware classification performance

To demonstrate the in situ hardware classification, we fabricated the peripheral circuit of the hardware neural network on a printed circuit board (PCB) using EC-RAM as the weights (see fig. S1 and Materials and Methods) such that every PCB represents one layer (including the ReLU activation function; see Supplementary Text 1) that can be cascaded to construct multilayer hardware neural networks. Figures S2 and S3A show the PCB and its block diagram, respectively, designed to operate as a single crossbar (network layer) of a 2 × 2 neural network (i.e., two inputs and outputs and a bias). This structure requires six cells (each consisting of an EC-RAM and transistor), of which four represent the weight values ( $w_{\mathrm{ij}}^{L}$ ) connected to the input voltages and two represent the bias ( $b_{j}^{L}$ ) connected to a constant voltage. In each cell, the gate of the EC-RAM is connected to the transistor to perform the multiplication for the update (see figs. S3, B to D, and S4). A current offset (using a potentiometer and an inverting operational amplifier parallel to the EC-RAM) is applied to both devices to achieve positive and negative conductance values (fig. S3, B to D). According to the mode of operation (MOO) (see fig. S3), the crossbar can operate in three modalities: forward (MOO = 1), backward (MOO = 2), and update (MOO = 3). In feedforward mode (fig. S3B), the output ( $I_{j}^{L}=\sum_{i=1}^{n}V_{i}^{L-1}\;w_{\mathrm{ji}}^{L}+b,$ with $w_{\mathrm{ji}}^{L}=G_{\mathrm{ji},\text{EC}-\text{RAM}}-G_{\mathrm{ji},\text{ref}}$ ) is converted to a voltage signal and passed through the ReLU activation function (φ). The ReLU activation function is implemented in hardware using a diode such that only positive output values go through (fig. S5)

$$\begin{array}{cc} \varphi \left(x\right)=\text{max}\left(0,x\right)=\left\{ \begin{array}{cc} 0, & x<0\\ x, & x\geq 0 \end{array}\right. & \varphi \prime \left(x\right)=\left\{ \begin{array}{cc} 0, & x<0\\ 1, & x\geq 0 \end{array}\right. \end{array}$$

During forward operation, a latch is controlled to store the gradient of the ReLU [φ′ (*x*)=0 or 1]. In the backward mode (fig. S3C), the backflowing error $\left[I_{i}^{\left(L-1\right)}=\sum_{j=1}^{m}V_{j}^{L}w_{ji}^{L}\right]$ travels in the opposite direction and is converted to a voltage multiplied by the stored gradient of the ReLU activation function (φ′). The third MOO, the update mode, and allows the board to multiply the feedforward and backward signal from the adjacent layers in situ to change the weight values accordingly (fig. S3D). The activation function of the output layer is a linear sigmoid, which can be implemented in hardware (*24*). Here, we implemented it in software since it only affects the last layer.

**Fig. 3.**
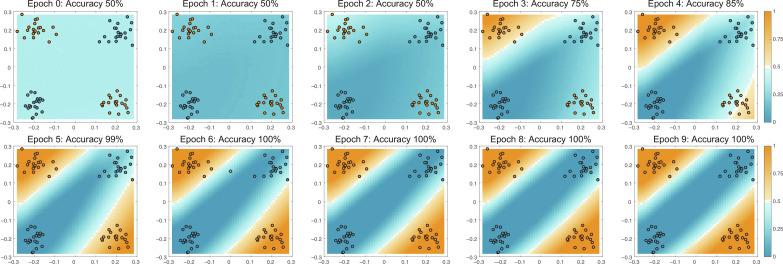
Classification domain with the decision boundaries for a two-layer classification after weight initialization (epoch 0) and after nine consecutive epochs. For the evaluation of the decision boundaries, the output values are recorded while sweeping over the input values from −0.3 to 0.3 V in a grid of 50 × 50. Blue and orange data points represent classes 0 and 1, respectively.

To demonstrate the progressive update approach in hardware, we cascade two crossbars to classify an exclusive or (XOR) dataset with a 2 × 2 × 1 neural network (see Fig. 2B; see Supplementary Text 2 and fig. S9 for classification of a single-layer hardware neural network). The first crossbar (2 × 2) is directly connected to the second crossbar (2 × 1) without any intermediate preprocessing software, and together, they form the total 2 × 2 × 1 neural network. While the entire neural network structure operates in hardware, the front end and back end of the network are controlled via software including input data sampling, error calculation, controlling of switches, and the hyperparameters. The dataset consists of data clusters, generated by a Gaussian distribution, *X*~𝒩(μ, σ^2^), with a given mean (μ) and SD (σ). Because this dataset can be visualized easily (two input features can be represented on a two-dimensional plane), it allows for better comparison and analysis on a qualitative level. The dataset contains four clusters of 20 data points with mean μ_1_ = ([−0.2, −0.2], [0.2,0.2]) for class 0 indicated in blue and μ_2_ = ([−0.2,0.2], [0.2, −0.2]) for class 1 indicated in orange and SD σ = 0.04. Figure 3 shows the decision boundaries for epochs between 0 and 9. One epoch represents one cycle through the feedforward and subsequent training cycles (progressively updating both layers from back to front) for each sample in the entire dataset. Before training, we initialize all weights close to 0 (see Fig. 2B, middle array) by discharging the gate of the EC-RAM and evaluate the network prediction across the solution domain. During training, an evaluation after every epoch is done as well. Epoch 0 represents the evaluation directly after initialization and shows that all the weights are close to 0 before training (see Fig. 3).

After the first two update cycles (epochs 1 and 2), all the weights have a value such that the output class is 0 across the entire solution domain (accuracy = 50%). After a few more epochs, the weights are further updated and reach an accuracy of 100% at epoch 6. The weights continue to update, leading to sharper decision boundaries but quickly saturate as the error, and thus the update, becomes 0 (see Fig. 2B, bottom array for the final weights after training). While the neural network structure, specifically the two neurons in the hidden layers, is sufficient to classify the XOR dataset, not all weight initializations lead to a global minimum and result in a 100% classification accuracy. Because of the inherent stochastic behavior of neural networks, training is sensitive to the configuration of hyperparameters such as weight initialization when using a network structure with the minimum required number of neurons. Depending on the random weight initialization values, training could get stuck in local minima. In fig. S10, we show another training process of the same neural network where the weights have different initialization values, which leads to a local minimum obtaining 75% accuracy. To reduce the probability of the neural network arriving in a local minimum, the number of hidden neurons can be increased. Still, the weight initialization step remains important and should be random and free from any biases caused by device and circuitry nonidealities such as offsets, gain errors, nonlinearities, and memory effects. In particular, in the beginning of the training when weight values are close to 0, these imperfections become dominant and can skew the results. Although those nonidealities cannot be completely avoided, great effort needs to be devoted to the design of the hardware neural network to mitigate their impact on the overall performance (see Supplementary Text 2). Even in software neural networks, fine tuning of the hyperparameters can have a large impact on the outcome. However, in software, many tools are available, such as normalization and regularization, whereas in hardware, this still requires manual optimization.

### Classification performance of parallel versus progressive update

While the progressive layer-by-layer update approach completely avoids the (digital) storage requirements and allows for efficient multiply-accumulate operations during backpropagation, the update values deviate slightly from the perfect update values as calculated by traditional gradient descent. Because the progressive layer-by-layer update moves from back to front after the error calculation, the last layer will get the ideal update step according to the gradient descent algorithm. However, the layers before the last one will get a slightly different backflowing error signal as the weights through which the error signal propagates are already updated. Although this deviation is unfavorable, generally, neural networks thrive when using large datasets with very small update steps. The weight update ( $∆w_{\mathrm{ij}}^{L}$ ) after evaluating a single data point is small enough that we assume that the value of the weight before and after the update is (almost) equal ( $w_{\mathrm{ij}}^{L}\approx w_{\mathrm{ij}}^{L}+∆w_{\mathrm{ij}}^{L}$ ). We thus expect that the impact of the imperfect backflowing error signal is minimal and does not significantly contribute to any accuracy loss. We verify this by simulating the performance of the proposed layer-by-layer backpropagation approach compared to the traditional parallel update method in software.

**Fig. 4.**
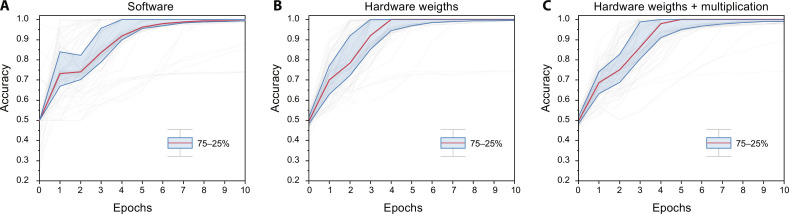
Accuracy comparison for software and hardware models. Training accuracy after every epoch for the (**A**) software model, (**B**) hardware model without transistor multiplication, and (**C**) hardware model with transistor model showing 100 training cycles (with randomized initial weight values). Blue area indicates the 25 to 75% boundaries, and the red line represents the median.

We used Python to model the behavior of the software and hardware neural network with the parallel and progressive gradient decent, respectively (see Supplementary Text 3). We chose a (2 × 7 × 1) neural network structure with two layers to classify a binary dataset, similar to the XOR dataset as shown in Fig. 3, with 100 data points per cluster μ_1_ = ([−0.3, −0.3], [0.3,0.3]), μ_2_ = ([−0.3,0.3], [0.3, −0.3]), and σ = 0.1. For robustness, we chose seven neurons in the hidden layer to counteract the probability of ending up in a local minimum as demonstrated with the hardware neural network (fig. S9). We use an ReLU and a linear sigmoid activation function for the hidden and output layers, respectively. The model for the software and hardware neural network are the same except for two important differences: First, the software model updates all layers in parallel (i.e., storing partial derivatives with respect to the weights in memory), while the model that is used to simulate the performance of the hardware neural network updates layers progressively, thus allowing slight deviations in error signals and consequently in the update steps. Second, the weight values in software can be programmed completely linear and exact, while the weights in the hardware model are retrieved from measurement data that include nonlinearity and noise (see Supplementary Text 4). All other parameters and processes such as learning rate, number of epochs, weight initialization, data preprocessing, network structure, activation functions, error function, and dataset are kept the same. To demonstrate the effect of the transistor multiplication, the hardware model allows us to perform the update multiplication either in software or with the experimental data of the transistor multiplication (fig. S4). Figure 4 shows the training accuracy after every epoch for the software and both hardware neural network models for 100 cycles. The performance strongly depends on the (randomized) initial weight values and varies cycle-to-cycle due to the inherent stochastic nature of the backpropagation algorithm. Both software and hardware models show a 100% classification accuracy after 10 epochs (for at least 75% of the runs), showing similar learning characteristics. Even with seven neurons in the hidden layer, still a few cycles ended up in a local minimum for all models.

**Fig. 5.**
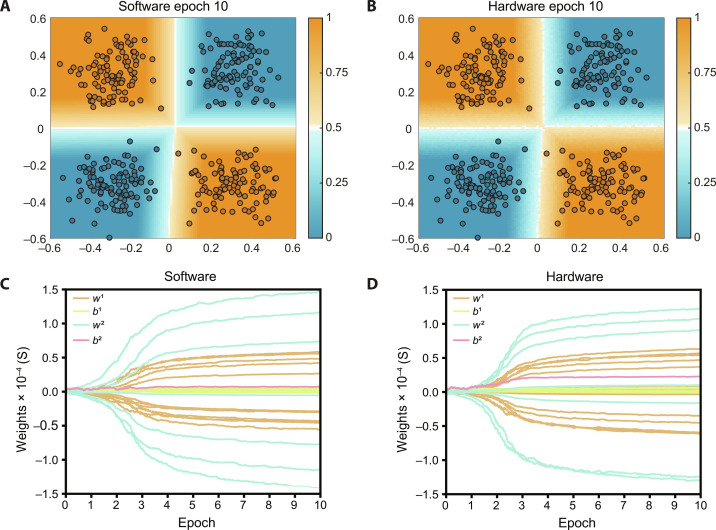
Decision boundaries and weight convergence comparison for XOR dataset using transistor multiplication in the update step. Discrimination boundary after 10 epochs for (**A**) software model and (**B**) hardware model. Weight convergence during 10 epochs for (**C**) software model and (**D**) hardware model, where *w*^(*i*)^ and *b*^(*i*)^ correspond to the weights and biases in the *i*th layer, respectively.

Figure 5 shows the discrimination boundaries and corresponding weight convergence, which demonstrates similar learning characteristics between the software model and the hardware model, using progressive gradient descent and transistor multiplication. When comparing the discrimination boundaries (Fig. 5, A and B), the noise (originating from read and write operation during the experiment; see Supplementary Text 4) in the hardware model is clearly visible, while the software model shows perfectly straight lines. However, this does not affect the accuracy since the noise level is much smaller than the input features. (In Supplementary Text 7, we further elaborate on noise considerations when scaling to larger neural networks.) Furthermore, when inspecting the convergence of the weights (Fig. 5, C and D), we see that the behavior of the weights in the software and hardware model are identical on a qualitative level and again show potential of the progressive gradient descent approach for hardware neural networks.

### Scalability

We investigated the scalability of progressive backpropagation in hardware for neural networks with more and larger layers and verify that the energy efficiency is orders of magnitude higher compared to traditional backpropagation (see Supplementary Text 6). The updating of each individual layer in our approach resembles the previously reported outer product weight update method (*13*, *14*, *28*) and thus can straightforwardly be scaled to larger size layers with more nodes. However, when expanding to a deeper neural network with more layers, some additional considerations can be taken into account to ensure good performance. Figure S14 shows that the classification result of progressive backpropagation for large neural networks solving the MNIST dataset is similar to traditional backpropagation. However, when solving more complex classification problems, it is important to choose a small learning rate to fulfill the condition $w_{\text{ij}}^{L}\approx w_{\text{ij}}^{L}+∆w_{\text{ij}}^{L}$ (see Supplementary Text 5). We found that implementing a learning rate decay, often used in software neural networks, is successful in keeping the update step small enough and the number of epochs limited (fig. S15). In addition, any stochasticity (or write noise) in the conductance state (weight) update across the array, regardless of the memristive technology used, should be low enough to keep satisfying the weight update condition above. We also explored the direction of progressive update and found that updating the layers in the forward direction (rather than the backward direction) can lead to faster convergence particularly when no learning rate decay is applied (fig. S16). Last, other software strategies such as batch training can be considered and there are various ways to implement this in hardware, however, at the cost of additional memory as discussed in Supplementary Text 8.

## DISCUSSION

We introduced an in situ backpropagation strategy that progressively updates neural network layers directly in hardware. This method allows us to calculate the partial derivative of the error with respect to the weight locally, thus bypassing the problematic requirement to store that information digitally or in hardware too. We executed training of a multilayer neural network using progressive gradient descent in hardware and demonstrated the classification of a two-layer hardware neural network by efficient matrix-vector multiplication of the update signal using a transistor at the gate of the EC-RAM devices. Through simulation, we have shown that propagating the error signal through an already updated layer has no impact on the classification accuracy for a two-layer neural network. We also show that, for larger neural networks, the impact can be made negligible by selecting small learning rates. The progressive backpropagation approach thus creates opportunities for in situ training of deep neural networks, harnessing the power of the well-established backpropagation algorithm without relying on pretrained software models or costly additional digital storage. This advancement enables substantial progress in intelligent computing systems, especially in contexts where online and continuous learning are crucial.

## MATERIALS AND METHODS

### EC-RAM fabrication

Cr (5 nm)/Au (50 nm) electrodes were patterned on a SiO*_x_* substrate using photolithography and the subsequent liftoff process of the negative photoresist (AZ nLOF 2035). After a surface treatment with the adhesion promoter Silane A-174, parylene C (~1.7 μm) was deposited on the substrate to electrically isolate the electrodes. A diluted Micro-90 (2% v/v in DI water) was spin-coated as an antiadhesive layer, and subsequently, a sacrificial second parylene C layer (~2 μm) was deposited. The EC-RAM channels, with a width of 200 μm and length of 500 μm, and contact pads were opened through successive photolithography (AZ10XT photoresist) and reactive ion etching steps (Nordson March RIE 1701). A Poly(3,4-ethylenedioxythiophene) polystyrene sulfonate (Hereaus, Clevios PH 1000) solution containing 6 vol % ethylene glycol (Sigma-Aldrich) to enhance the morphology and 1 vol % (3-glycidyloxypropyl)trimethoxysilane (Sigma-Aldrich) as a cross-linking agent to improve mechanical stability was filtered through a 0.45-μm polytetrafluoroethylene filter and spin-coated on the patterned substrates (1500 rpm for 1 min) and baked at 120°C for 10 min. Thereafter, the substrate was gently rinsed in deionized water to eliminate residual contents and subsequently dried at 120°C for 60 min after which the sacrificial parylene C layer was removed. The ion gel solution was prepared by dissolving ionic liquid 1-Ethyl-3-methylimidazolium bis(trifluoromethylsulfonyl)imide and poly(vinylidene fluoride-*co*-hexafluoropropylene) (4:1 w/w) in acetone (17.6 wt % ionic liquid, 4.4 wt % polymer, and 78 wt % solvent) and stirred at 40°C for at least 30 min, after which it was dropcasted on the EC-RAM substrate to define the solid electrolyte.

## Supplementary Materials

### This PDF file includes:

Supplementary Texts S1 to S8 Figs. S1 to S19 Table S1 References
