# Supplementary material for: Hardware implementation of backpropagation using progressive gradient descent for in situ training of multilayer neural networks
Source: Sci Adv. 2024 Jul 12;10(28):eado8999. doi: 10.1126/sciadv.ado8999 (PMC11244533; doi:10.1126/sciadv.ado8999)
Supplement: Supplementary file 1 — Supplementary Texts S1 to S8 Figs. S1 to S19 Table S1 References [file sciadv.ado8999_sm.pdf]

Science Advances
AAAS

Supplementary Materials for

Hardware implementation of backpropagation using progressive gradient descent for in situ training of multilayer neural networks

Eveline R. W. van Doremaele *et al.*

Corresponding author: Marco Fattori, m.fattori@tue.nl; Yoeri van de Burgt, y.b.v.d.burgt@tue.nl

Sci. Adv. **10**, eado8999 (2024)
DOI: 10.1126/sciadv.ado8999

This PDF file includes:

Supplementary Texts 1 to 8
Figs. S1 to S19
Table S1
References

Supplementary Text

Supplementary Text 1: Hardware implementation of gradient descent

Transistor multiplication

For the multiplication of the feedforward and backflowing error (**Fig. S 3D**) we use a normally semi-open JFET transistor operating in the linear region where the output current I_D is proportional to: $I_D \propto V_G V_D \Leftrightarrow |V_G| \gg |V_D|$. We pair the transistor with a 100 Ω resistor to correct for the offset such that its conductance is zero when $V_G = 0$V. **Fig. S4** shows the output current as a result of the voltage multiplication. The linearity of this multiplication directly impacts the weight update and thus the network performance.

Hardware ReLU

To implement the rectified linear unit (ReLU) in hardware the current output from the crossbar column is first converted to a voltage by a Trans-Impedance Amplifier (TIA) with a resistive feedback (R1), before it is fed to a diode (see **Fig. S5**). When the diode is operated in forward bias a voltage drop of 80mV should be accounted across the device. This forward voltage introduces an offset in the value of the ReLU output. To reduce the impact of this offset, the output swing of the TIA is maximized to a ±5V by proper sizing of the resistor R1. Indeed, the magnitude of the amplification is determined by the resistor R1 (see **Fig. S5**), while the change of polarity is introduced by the inverting configuration of the TIA. To correct for this the diode is reversed in the circuit, allowing only negative values to pass (that correspond to a positive output). The diode is followed by another scaling circuit, to reduce the voltage output back to a ± 300 mV range that is a suitable input level for the next layer. This attenuation is determined by the ratio R2/R3 (see **Fig. S5**). The gradient for the backpropagation is computed using a comparator circuit followed by a latch to store the binary result of the comparison. A voltage divider is connected to the non-inverting terminal of the comparator. In this way, the potentiometer (R4) can be tuned to set the switching voltage of the comparator at 0V, representing the gradient of a ReLU (see **Fig. S5**).

Weight modulation EC-RAM

We implemented a current source (**Fig. S6A**) in front of the EC-RAM gate to scale the output current from the transistor (the result from the forward and backward multiplication) to the desired update steps. The use of the current source allows for linear update steps and facilitates to set the maximum and minimum conductance values by balancing the resistors to prevent device damage. **Fig. S6B** shows the conductance modulation by applying alternating pulse trains of positive and negative voltage to the input of the current source. When increasing the number of pulses, we can see that the maximum and minimum values set by the current source are reached (**Fig. S6C)**.

Weight update in the hardware neural network.

As shown in **Fig. 1B** the weight update is calculated by applying the feedforward and the backpropagation signal at the same time. Previous research demonstrated the calculation of the update by executing the outer product in hardware(*13*, *14*). Our hardware on the other hand, allows signals to travel through all the layers sequentially, while updating the layers immediately, and therefore preventing the need to store the input and error signals. The feedforward signal (a^{L-2}) travels downstream through the network until the layer ($L-1$) that requires updating, according to:

$$z^L = \Sigma w^L a^{L-1} + b$$
$$a^L = \varphi(z^L)$$

$$z^{L-2} = \Sigma w^{L-2} a^{L-3} + b$$
$$a^{L-2} = \varphi(z^{L-2})$$

The error propagates backward (upstream) through the network until the layer $(L-1)$ and is therefore multiplied by the weights (w^L) in layer (L). This can be calculated using the chain rule:

$$\frac{\partial E}{\partial w^L} = \frac{\partial z^L}{\partial w^L}\frac{\partial a^L}{\partial z^L}\frac{\partial E}{\partial a^L} = a^{L-1} \cdot \varphi'^{L} \cdot 1$$

$$\frac{\partial E}{\partial w^{L-1}} = \frac{\partial z^{L-1}}{\partial w^{L-1}}\frac{\partial a^{L-1}}{\partial z^{L-1}}\frac{\partial z^L}{\partial a^{L-1}}\frac{\partial a^L}{\partial z^L}\frac{\partial E}{\partial a^L} = a^{L-2} \cdot \varphi'^{L-1} \cdot w^L \cdot \varphi'^{L} \cdot 1$$

The network in **Fig. 1** consists of 2 output neurons leading to $\vec{E} = E_1 + E_2$. As such the update of the weights in layer $(L-1)$ are the combined effort of the two errors:

$$\Delta w^{L-1} = -\eta \frac{\partial \vec{E}}{\partial w^{L-1}} \vec{E} = -\eta \frac{\partial E_1}{\partial w^{L-1}} E_1 - \eta \frac{\partial E_2}{\partial w^{L-1}} E_2$$

$$\Delta w^{L-1} = -\eta \cdot a^{L-2} \cdot \varphi'^{L-1} \cdot {w_1}^L \cdot \varphi'^{L} \cdot E_1 - \eta \cdot a^{L-2} \cdot \varphi'^{L-1} \cdot {w_2}^L \cdot \varphi'^{L} \cdot E_2$$

$$\Delta w^{L-1} = -\eta \cdot a^{L-2} \cdot (\, \varphi'^{L-1} \cdot {w_1}^L \cdot \varphi'^{L} \cdot E_1 + \varphi'^{L-1} \cdot {w_2}^L \cdot \varphi'^{L} \cdot E_2)$$

$a^{L-2} = \varphi(z^{L-2})$ is the forward signal and $(\, \varphi'^{L-1} \cdot {w_1}^L \cdot \varphi'^{L} \cdot E_1)$ and $(\varphi'^{L-1} \cdot {w_2}^L \cdot \varphi'^{L} \cdot E_2)$ are the backflowing error signals originating from the errors at the two neurons, which can travel in parallel through the network to update the weights w^{L-1}.

Supplementary Text 2: Hardware neural network design and testing.

The PCB is able to execute all computations (except for the generation of input data and the error calculation) depending on the mode of operation (MOO) and is controlled by an NI multifunction I/O device (DAQ) using MATLAB software (see **Fig. S7** and **Table S1)**. From the perspective of the NI board, the first 5 channels are analogue outputs (AO), responsible for input features and backflowing errors in the crossbar. Channel 6 through 8 are connected to digital outputs (DO) to control the mode of operation (feedforward, feed backward or update), referring to the progressive backpropagation (**Fig. 1C**). Channels 9 and 10 are also digital outputs (DO), which are used to store the gradient of the activation function (0 or 1) during feedforward and to apply the update on the gate of the EC-RAM, respectively. The last two channels are analogue inputs (AI) used to measure the output of the crossbar. Note that each additional crossbar comes with the same channel configuration where the crossbar output can be connected directly to the input channels of the next crossbar in order to create a multi-layer neural network. To classify an

XOR dataset, 2 crossbars have to be connected in series, representing a 2×2×1 hardware neural network, and progressively need to change their mode of operation. **Figure S8** shows the control sequence of the 2-layer hardware neural network. During the first part of the signal sequence, both crossbars are set to the forward mode in order to apply the input signals which will result in a network prediction, where after the gradient values for the ReLU activation functions are stored. The second and third part of the signal sequence are responsible for updating crossbar 2 (CB2) and crossbar 1 (CB1), respectively. The error, calculated in the first sequence, is propagated backwards through the crossbars by applying the correct mode of operation. The update channels can then be used to apply the update to each crossbar, using the signal duration as learning rate.

Single layer classification

Here we demonstrate the behavior of a single crossbar using the transistor multiplication to update the weights, by replicating a single layer (2×1) neural network using only 1 output column of a single crossbar with 3 weights. The dataset $X \sim N(\mu, 0)$ consist of two clusters with 2 data points with $\mu_1 = (-0.3, 0.3)$ and $\mu_2 = (0.3, -0.3)$ for class 1 (orange) and 0 (blue), respectively. **Figure S9** shows the decision boundaries for Epoch 0, 1 and 10.

Epoch 0 represents the classification output when the weights are randomly initialized, and the network is not trained. We can clearly see that the values of the weights are close to zero, as there the decision boundary is curved. Note that a single layer neural network (with 2 weights and a bias) only allows for a linear separation between classes. The curved decision boundary is the effect of noise on the weights. Every epoch the network runs through all datapoints and updates the weights accordingly. From the decision boundaries we can clearly see the update mechanism works well since the accuracy of epoch 1 and 10 is 100% for this dataset. We also see that the contribution of noise is reduced after only 1 epoch. After 10 epochs the network shows an almost perfect classification boundary. The classification using 1-layer shows that the update method using the transistor multiplication works as expected. Note that it only allows us to demonstrate the forward and update mode of the crossbar (and not the backflowing error).

Impact weight initialization

Figure S10 shows the decision boundaries for various epochs between 0 and 10. Epoch 0 shows that all the weights are close to zero before training. The dataset consists of 4 clusters with $\sigma = 0$ and 10 datapoints per cluster with standard deviation $\sigma = 0$

After the first update cycle (epoch 1) all the weights have a value such that the output is always zero (accuracy = 50%). After a few more epochs the weights are updated and reach an accuracy of 75% at epoch 6. The weights continue to update but after 10 epochs the output accuracy remains 75% and the network is unable to correctly classify the data points at (-0.3,0.3) as 1. This training example demonstrates a neural network that is stuck in a local minimum. As discussed before, when dealing with a small number of neurons the probability that the network encounters a local minimum is significant and the success of the neural network training strongly depends on the weight initialization. In the simulation we decreased this probability by increasing the number of neurons in the hidden layer to 7 to increase the likelihood that the network can create a combination of useful weights. Since we are limited by the dimensions of the designed crossbar, we can only use 2 neurons for this demonstration.

Hardware classification performance.

Successful operation of neural networks, both hardware as well as software, require fine tuning of the hyperparameters. The first consideration is signal strength throughout multiple layers (in software referred to as vanishing signals). While the input to the first layer is normalized (in software) the output of the first layer is orders of magnitude lower due to the multiplication of the weight value (~0.1 mS range). In software the output signal in between layers can be normalized, while in hardware we implemented an amplification factor that requires careful tuning. If the amplification factor is too small the signals throughout the layers will vanish and only noise will have an impact on the system. On the other hand, if the amplification factor is too large the devices will break down. Furthermore, since the output value is an accumulation of all the currents in the column the amplification depends on the number of neurons and on the conductance range of each EC-RAM. The same is true for the backflowing error, which vanishes throughout the multi-layer neural network if the amplification between layers is too small. The output of the backflowing error is accumulated over all currents in a row, and its optimal amplification can thus be different from the forward signal amplification. Another parameter that requires optimization is the activation function, in particular the one of the output layers. We have implemented a linear sigmoid that scales the output values to a prediction with a certain probability and allows to differentiate between different error strengths. We can define its boundaries such that the error of correct predictions contributes more or less to the total error by increasing or decreasing the boundaries of the sigmoid, respectively. For example, by decreasing the boundaries the slope of the sigmoid increases which means that small variations in the output results in a large impact on the error while an output voltage (larger than defined boundary), results in a maximum output and if this prediction is correct, does not contribute to the error. If the boundary is set too small (set to zero it becomes a Heaviside step function), the error is unable to differentiate between the output since after the activation function all outputs will be binary, resulting in either a maximum error or no error value.

Supplementary Text 3: Hardware neural network simulation

The simulation of the hardware neural network is performed with Python, substituting the floating-point precision weights of a software neural network with a discrete set of conductance states extrapolated from data measured on the same EC-RAM devices used for experimental validation (as depicted in **Fig. S11**). In particular, the EC-RAM data of the conductance modulation (**Fig. S6B**-**C**) is divided in a reset (potentiation) and set (depression) file containing each 128 (or 200) states for 15 cycles. A conductance state is defined as the average conductance of 10 data points per state, while their standard deviation is used to model a (normally distributed) read noise (see **Fig. S 12**). The average gradient for potentiation and depression per conductance state is calculated and interpolated for conductance values in between. Since the update (change in conductance) is proportional to the time and the voltage amplitude, the average gradient is divided by the pulse duration (20ms) and update voltage (±50mV). The write noise is determined by the standard deviation between cycles. **Figure S13** shows the cumulative distribution function (cdf) of the mean conductance gradient versus conductance for potentiation and depression of the conductance values. It shows that the updates are symmetric but slightly non-linear in the conductance range $C = 7 \cdot 10^{-4}$ S to $C = 11 \cdot 10^{-4}$ S. This is caused by the drain voltage ($V_D =$ 0.1V) during the measurement. Below conductance values of $C = 7 \cdot 10^{-4}$ S we see the capping effect of the current source indicating conductance values close to its boundary preventing further updates towards low conductance values. The offset value (to realize negative weight values) is

determined by conductance of the channel when the gate of the EC-RAM is fully discharged. When set and reset are asymmetric, it is recommended to determine the offset value by the intersection of the increasing and decreasing gradient, preventing biases introduced by different update values for increasing and decreasing states.

Supplementary Text 4: Noise calculation for hardware neural network simulation.

The model considers two contributions of noise, read and write noise, which are both derived from experimental data containing the conductance modulation of the EC-RAMs (**Fig. 6B-C)** for potentiation and depression for 15 cycles. The read noise is based on the standard deviation of 10 data points per state (see **Fig. S12**) leading to a read noise value for all states and cycles. These values are averaged over all 128 states of 14 cycles (omitting the data of the first cycle). The model uses a single read noise value by averaging the read noise of both potentiation and depression resulting in a value of $\sigma_{rn} = 9.5 \cdot 10^{-8}$. The write noise is determined by the standard deviation of the conductance gradient divided by the duration of 20ms and update voltage of $\pm$50mV for the last 14 cycles ($SV^{-1}s^{-1}$) and results in a write noise of $\sigma_{wn} = 2.2 \cdot 10^{-5}$ and $\sigma_{wn} = 3.4 \cdot 10^{-5}$ for potentiation (increasing conductance) and depression (decreasing conductance) respectively. The model assumes that both read and write noise values are constant across the conductance and voltage domains.

Supplementary Text 5: Algorithmic scalability of progressive backpropagation

To test the scalability of progressive backpropagation additional simulations are performed. We compare our approach with the traditional software implementation of the backpropagation algorithm on the MNIST dataset consisting of 70,000 labeled images of handwritten digits (*31*). Training is done using stochastic gradient descent with a batch size equal to 1, to mimic hardware operation settings and to limit memory usage. Unless otherwise indicated the simulations use a fully connected 784×125×25×10 network. Its input layer contains 784 input neurons corresponding to the 28×28 pixels of the image. Its output layer contains 10 output neurons representing the numbers 0 to 9. The two hidden layers vary throughout the simulations. The models for progressive and traditional backpropagation use a ReLU and a Linear Sigmoid activation function for the hidden and output layers, respectively. The error is calculated as the difference between the output and the target. Both models are the same except for update mechanism: The traditional model updates all layers in parallel (i.e. storing partial derivatives with respect to the weights in memory), while the model for progressive backpropagation updates layers progressively, thus allowing slight deviations in error signals and consequently in the update steps. Therefore, the update values of all the layers, except for the last, are slightly different. If the weight update (Δw_{ij}^L) is small enough, we assume that the value of the weight before and after the update is (almost) equal ($w_{ij}^L \approx w_{ij}^L + \Delta w_{ij}^L$) and expect a limited impact of the imperfect backflowing error signal. The weight update value is calculated according to: $\Delta w_{ij}^L = -\eta \frac{\partial \vec{E}}{\partial w_{ij}^L} \vec{E}$ and thus depends on the learning rate. We therefore simulate the accuracy for various learning rates from 0.005 to 0.0005. We also vary the number of hidden layers and neurons in these layers to represent larger neural networks that can deal with more complex problems. In **Fig. S14** the result of the simulation is shown that demonstrates the comparison between the traditional and progressive

backpropagation for a range of neural network architectures (varying in the composition of hidden layers) and learning rates. The numbers within the heatmap represent the number of epochs that is required until the stopping criterium is met. We consider three stopping criteria. The first criterium is when the simulation accuracy converges to 97% which represents a neural network with good hyperparameters (the dark green areas in **Fig. S14**). The second criterium considers a network that does not converge and drops in accuracy and stops the simulation when the loss of an epoch becomes larger than three times the best loss the simulation has seen (red areas in **Fig. S14**). The last criterium stops after 350 epochs in case the network did not drop in accuracy but also did not yet converge to 97% (the light green areas in **Fig. S14**).

From **Fig. S14** it can be seen that smaller neural network architectures, such as the case without hidden layers (No HL) and the case with 1 hidden layer (1 HL) with 25 neurons, do not converge to 97% accuracy within 350 epochs both for the traditional and progressive approach. When adding more neurons to the hidden layer, 50 and 100 neurons, the stopping criterium of 97% accuracy is met. We see that the number of epochs required to reach 97% accuracy decreases with increasing number of neurons and hidden layers in a similar way for both backpropagation methods. However, when increasing the network complexity (to two hidden layers), the progressive backpropagation approach for a learning rate of 0.005 shows a sudden divergence of the weights causing a drop in the accuracy with a loss that keeps increasing. We believe that the magnitude of the update step to allow for the assumption $w_{ij}^L \approx w_{ij}^L + \Delta w_{ij}^L$ depends on the complexity of the problem and the corresponding complexity of the neural network architecture required to solve the problem. We therefore see that when reducing the learning rate (e.g. 0.001 and 0.0005) for the same problem and neural network architecture, the model is able to converge to 97%. By reducing the learning rate, the computation time increases. In **Fig. S15** this can be seen by the increasing number of epochs. On the other hand, in the beginning of training a neural network the importance of a small learning rate is limited. A technique, common in software neural network training called learning rate decay, is able to leverage the small learning rate when the weight values are close to their solution and uses a larger learning rate in the beginning of training that allows for faster convergence. In **Fig. S15** we show the accuracy for different learning rates after every epoch for both the traditional and progressive backpropagation methods. This neural network consists of two hidden layers with 125 and 25 neurons. The higher learning rates 0.005 and 0.003 show a drop in accuracy for the progressive backpropagation model. The smaller learning rate such as 0.001 shows an almost identical behavior compared with the traditional backpropagation method, indicating that the error in the weight update is negligible. **Figure S15** also shows that a gradual learning rate decay can be implemented that balances a large learning rate (0.003) in the beginning of training and a small learning rate (0.001) when the update step becomes critical. Every epoch the learning rate is gradually reduced by 5% until a learning rate of 0.001.

Alternatively, the direction of updating the layers progressively can also be changed. Instead of starting with the last layer and progressively update the layers upstream (backward progressive), the updates can start at the front and progressively update the layers downstream (forward progressive). Until now we only considered backward progressive as the proposed method to implement backpropagation in hardware. While in the case of backward progressive gradient descent the previously updated weights cause an error in the backflowing error signal, the error in the case of forward progressive gradient descent occurs in the forward propagating input signal. For a two-layer neural network (as demonstrated both in hardware and software) as well as for a larger neural network with a small (enough) learning rate, the updating error due to the deviating backflowing signal does not lead to accuracy loss. However, some of the cases with more hidden

layers and a high learning rate (see **Fig. S14**) showed a drop in accuracy. In these cases, it can be beneficial to update the layers progressively in a forward direction. In **Fig. S16** we show that with the forward progressive updating method the neural network with two hidden layers (125 and 25 neurons) and a learning rate of 0.003 is able to converge to 97% similar to the traditional backpropagation method whereas the backward progressive method failed due to the high learning rate. We hypothesize that the impact of a slightly wrong input signal is less than the impact of a slightly wrong error signal. In depth study is required to unveil the true cause. With these simulations we show that the proposed progressive backpropagation method can scale to larger neural networks to solve more complex problems. When optimizing hyperparameters such as the learning rate, it is important to choose a learning rate that is small (depending on the complexity of the problem and the neural network architecture) or a gradual learning rate decay can be considered. We also demonstrated that the forward direction of the progressive backpropagation can help to prevent the sudden drop in accuracy. Note that a systematic study on the direction and the learning rate is required to draw conclusions on their actual impact and exact relationship.

Overall, our approach eliminates memory requirements during the training of neuromorphic devices using backpropagation. This allows for *in situ* training while achieving convergence times comparable to the traditional backpropagation method.

Supplementary Text 6: Hardware scalability of progressive backpropagation

When scaling the proposed hardware neural network to larger architectures, during backpropagation the most demanding task in terms of energy and latency, is the update of the weights. The update of a single weight depends on the time required to (dis)charge the EC-RAM, which is dependent on the current source and learning rate. However, the weights of each layer can be updated all in parallel, leading to an overall latency for the training of one datapoint which scales linearly with the number of layers. In terms of energy, the update of each individual weight is dependent on the charge to be injected in the EC-RAM and the energy cost of a multiply-accumulate (MAC) operation, which carry the backflowing error and feed forward signals through the crossbars of the other layers. Moreover, in software the data required for the training has to be transferred between the memory and computational units while in hardware, thanks to the characteristics of these neuromorphic devices, the MAC operation and storage of the results occur simultaneously.

When scaling our progressive backpropagation approach to large-scale neural networks (e.g. with the 784×125×25×10 architecture) a similar hardware implementation could still be used. Clearly, to implement such system, a Si-CMOS Integrated Circuit (IC) solution is needed, and the EC-RAM should be processed e.g., on the back-end of the chip. Depending on the capabilities of the integration process, the time duration of the forward/backward propagation and weight update phases could be impacted by the interfacing circuits. Indeed, as shown in the literature organic EC-RAM can achieve writing/reading speeds in the ns range (*7, 32*). On the contrary, operating peripheral and interfacing electronics at similar speeds might be challenging mostly due to parasitic effects introduced by the integration of the EC-RAM. Further studies are required to assess this, starting from the development of the integration process and the synthesis of the interfacing electronics in a custom IC. For these reasons, we have discarded here the contribution of the peripheral circuits when estimating the energy required to train a network implemented in hardware using neuromorphic devices and the progressive error backpropagation method. This energy consumption is then compared to the one required to train the same network in software

(namely using a von Neumann architecture (*33*) and a conventional backpropagation approach). It should also be mentioned that despite several strategies have been proposed in literature (*34*) to assess and compare the energy costs of the training and inference operations of neural networks, there is no standardized way of benchmarking this performance. Therefore, we propose here to benchmark the training of a neural network, using as a main indicator the energy consumption required to perform MAC operations and memory accesses necessary to compute and backpropagate the error and update the weights.

In a software representation, there are $784 \cdot 125 + 125 \cdot 25 + 25 \cdot 10 = 101375$ weights and thus an equivalent number of MAC operations required for inference. Moreover, the backpropagation, which includes the weight update and the backward pass of the error requires the same number of MACs with an additional $125 \cdot 25 + 25 \cdot 10$ MACs, leading to a total number of 206125 MACs (including the inference and error backpropagation for one data point). Assuming an energy of 3.7 pJ/MACs (32-bit floating operation (*35*)), for the computation of a single data point (e.g. an image), the software backpropagation requires 763 nJ. Even more significant is the energy cost required by one memory access which ranges between 5 pJ and 640 pJ for a 32-bit coefficient stored in on-chip Static Random Access Memory (SRAM) and off-chip Dynamic Random Access Memory (DRAM), respectively (*35*). Since large neural networks do not fit in on-chip storage, more costly DRAM accesses need to be accounted for. Hence, during inference the values need to be stored to memory and during the update of each weight in the neural network these values need to be retrieved from memory. Therefore, the energy required to access DRAM, for inference and the training on a single data point reads $E_{SW-TR} = 132$ µJ. For completeness, it should be noted that the energy costs of the writing operations required to update all the coefficient in DRAM is still excluded in this estimation.

To allow for the integration in hardware of the considered neural network example and thus its fabrication on the silicon back-end we should assume a downscaling in area of our organic EC-RAM devices of at least a factor ~100. However, it is not realistic without any further experimental verification to assume that our EC-RAM electrical parameters would linearly scale with the area of the device. Therefore, to estimate the energy consumption of a relatively large neural network implemented in hardware and compatible with our backpropagation algorithm, we have considered here the downscaled EC-RAM devices proposed in (*7*). Indeed, these devices feature channel areas as low as 45×15 µm^2, that can be programmed with 20 ns pulses, resulting in an energy consumption of $E_{ECRAM-WR} = 80$ fJ per write operation (*7*). In order to perform a weight update step, our approach relies on the tuning of the EC-RAM conductance by injecting (or extracting) of multiple "charge packets" over time. This can be achieved e.g., using a duty-cycled constant current source per device (*32*). In this way, the charge in these packets can be controlled either by changing the nominal value of the current source or by modifying its duty-cycle (**Fig. S6**). Since the EC-RAM writing is current-driven, no additional significant amount of energy needs to be spent by the driving circuit to perform the update. Therefore, we can estimate that in a hardware implementation the energy required to update the weights using the progressive error backpropagation method is equal to the number of weights multiplied by the EC-RAM writing energy $E_{ECRAM-WR}$, leading to $E_{HW-UP} = 8.1$ nJ for a single data point. However, to this estimation, one should also consider the energy spent for the backpropagation of the error along the layers of the neural network. Indeed, in order to propagate the error and locally compute the update contribution for each weight, several MAC operations need to be performed using the EC-RAM, leading to an additional energy cost defined here as E_{HW-MAC}. It can be calculated that in the considered neural network (784×125×25×10) the progressive error backpropagation requires a total amount of 304125 MAC operations (for inference and backpropagation including the

forward and backflowing error signals). Since the conductance of the EC-RAMs in (*14*) can be linearly tuned between 30 and 60 μS, an average conductance value of g_m=45 μS is here selected for the estimation of a MAC energy cost. Moreover, in (*14*) MAC operations can be executed in about 300 ns using a signal amplitude of V_{IN}=300 mV. Note that this (prolonged) time is chosen to ensure a stable reading of the MAC while ideally this time decreases to the update time step (i.e. 20 ns). The time required by the MAC operation, should be further extended by the update time of 20 ns since the multiplication values need to be available during the entire the update phase. Therefore, the time of a MAC operation is here considered to be T_{MAC}=320 ns. Thus, the energy required for each MAC operation is estimated to be:

$$E_{ECRAM-MAC} = g_m \cdot T_{MAC} \cdot V_{IN}^2 = 1.3 \text{ pJ}$$

For the hardware implementation case, the total energy required to train this specific neural network on a single data point can be calculated as follows:

$$E_{HW-TR} = E_{HW-UP} + E_{HW-MAC} = (E_{ECRAM-WR} + E_{ECRAM-MAC} \cdot N) \cdot w_n$$

Where E_{HW-TR} is the total energy consumption per datapoint, N is the number of layers and w_n is the number of weights in the network. For the network under consideration (784×125×25×10) this leads to an energy consumption of $E_{HW-TR} = 403.1$ nJ per datapoint, which is order of magnitudes lower than the one needed for training a neural network in software executed on a Von Neumann architecture. For completeness, it should be mentioned that the previous result does not account for the energy consumption of the transistor-based signal multiplication required for each weight during backpropagation (**Fig. S4A**). The transistor-based multiplication can be rather efficient thanks to the advantages of Si-CMOS technologies and therefore, its contribution can be neglected here.

The proposed progressive backpropagation method enables in-hardware training of neural networks using any generic 3-terminal neuromorphic technology. Furthermore, the provided comparison reveals that the neuromorphic computing approach has the potential to achieve training of relatively large neural networks with significantly higher energy-efficiency compared to e.g., software training based on classical von Neumann architectures. It is important to remark that a fairer comparison would require to also include the energy consumption of all the peripheral circuits required to operate the neural network in forward, backward and update mode. These circuitries are mostly based on digital logic (and require limited number of gates), whose functions are rather efficient in advanced silicon technology nodes. Among the peripheral modules, the most power-hungry circuits remain the Trans-Impedance Amplifiers (TIAs), required to collect the currents of the different synapses as well as the unity-gain buffer required to apply the current bias compensation to the EC-RAM. The very same circuits are needed to operate each crossbar and are re-used for both the forward and backward propagation. Despite the energy-efficient and relatively high-speed solutions that can be achieved (e.g., using inverter-based topologies), the energy consumption of the peripheral circuits strongly depends on the Si-CMOS technology utilized, and cannot be reliably estimated prior to their implementation.

Supplementary Text 7: Noise scalability for progressive backpropagation

Regardless of the implementation of backpropagation all neural network architectures in hardware have to deal with the non-idealities such as noise. Its impact on the performance depends on the nature of the noise. In most cases, the noise can be assumed to follow a Gaussian distribution

where the mean is zero. In this case, all contributions of the noise will cancel out during the training when the size of the dataset and the number of epochs are sufficiently large. If the noise has a non-zero mean, the signals in the system will experience a one-directional offset. The spread, or the standard deviation, of the noise, (especially the read noise) directly impacts the number of states and therefore, the resolution of the neural network and the sharpness of the decision boundaries. Complex problems require weight values with high precision in order correctly discriminate between classes. Moreover, the read noise can affect the performance specific to certain applications. One-time-inference cases (which could be vital for deployment of the network) can be impacted by the spread since multiplying potential outliers in the noise distributions can be amplified. This could cause imperfect readings of the output where the frequency of this occurrence will depend on initial distribution spread and network size. The impact during training for both read and write noise is limited, since neural networks process a large amount of data the deviation caused by the noise will always approximate the mean value, thus cancelling out the spread of the noise. We performed a simulation adding a noise value with a zero mean and varying standard deviation to both the read and write operations in a 784×120×50×10 neural network. **Figure S17** shows that the progressive backpropagation method (in both backward and forward direction) behaves similarly to the traditional method when read noise is present. Again, we see that the backward progressive approach achieves lower accuracies for high learning rates (discussed in Supplementary Text 5). However, when the standard deviation of the noise increases, we observe that the accuracy for high learning rates improves. Although this needs to be investigated further, it could be the case that the read noise creates more randomization in the backpropagation algorithm improving the chance of convergence when the condition of $w_{ij}^{L} \approx w_{ij}^{L} + \Delta w_{ij}^{L}$ is not met. Randomization (or adding noise to signals) is a strategy that can be used to deal with local phenomena such as local minima. This observation would suggest that the occurrence of divergence in the progressive backpropagation algorithm is an effect that is happening locally. For high read noise standard deviations all backpropagation methods demonstrate a similar poor performance that can be attributed to the reduced number of available states. The impact of the write noise on the performance of the progressive backpropagation is significantly less (see **Fig. S18**). The write noise only contributes to the deviation in the calculated update step (according to the algorithm) and the actual update. Therefore, this property is completely independent of the method given that the mean of the noise is equal to zero, the average of the actual update (direction and magnitude) will be equal to the average of the calculated update step. As shown in **Fig. S18**, the impact of the write noise on the traditional backpropagation and progressive backpropagation are equivalent. However, it is important to note that in both cases the training time could drastically increase when the write noise is bigger than the roughness of the loss landscape. Overall, we can conclude that the impact of noise in a hardware neural network does not depend on the method of backpropagation.

Supplementary Text 8: (Mini)-Batch Gradient Descent

When expanding to larger networks, the implementation of training in batches can become relevant. (Mini)-Batch Gradient Descent (BGD) is a method that averages the update step over a given batch of datapoints such that the direction and magnitude of the updates are a more accurate representation of the Gradient Descent (GD) algorithm, while still leveraging the speed of Stochastic Gradient Descent (SGD). In order to update the weights according to the given batch, the update step should be averaged over the number of datapoints within the given batch and

therefore, additional memory is required. Based on previous research we consider two methods to realize (mini)-batch gradient descent in hardware using analogue memory.(*4*)

Both methods require a memory component such as a capacitor based short term memory cell. The first approach stores the update values for all datapoints of a batch.(*4*) This memory cell could potentially be implemented within our hardware neural network connected to the current source and the gate switch, where electrical charge is stored as analogue memory. When the batch is completed, all EC-RAMs will be updated simultaneously and only once (with the combined update values of the batch). Consequently, since the update values of all layers are calculated before any weights are updated, there is no error in the update value that originated from the progressive backpropagation. Furthermore, it reduces the amount of write cycles of the EC-RAMs which can enhance its lifespan. However, enabling batch training according to this approach, requires a large amount of memory cells scaling with $O(n^2)$, with n number of inputs/outputs, which will potentially counteract the performance gain from hardware neural networks.(*36*) The complete cell for this batch training approach requires, besides the additional memory component, two more switches and requires the current source to be used for both storing the update values and updating the EC-RAMs (see **Fig. S19**). It also introduces an extra mode of operation (MOO): Forward mode, backward mode, batch-store mode (previously update mode see **Fig. S3D**), and batch-update mode. Alternatively, a second way of implementing batch training in hardware neural networks places a memory component at the inputs and outputs rather than in the cell. All the inputs and outputs of the layers are stored for every datapoint in the batch, resulting in analogue memory components that scale with $O(2n \cdot batch\ size)$. Similar to the first batch training method, there is no error due to already update weights, however unlike the first, this method does not reduce the amount of write operations and its scalability depends on the batch size.

Depending on the size of the neural network and the batch size one of the two approaches might be better in terms of number of additional components. The cost of introducing the additional (memory) components with respect to the gain of the hardware neural network performance is not investigated and should be considered to determine the best approach for a specific problem.

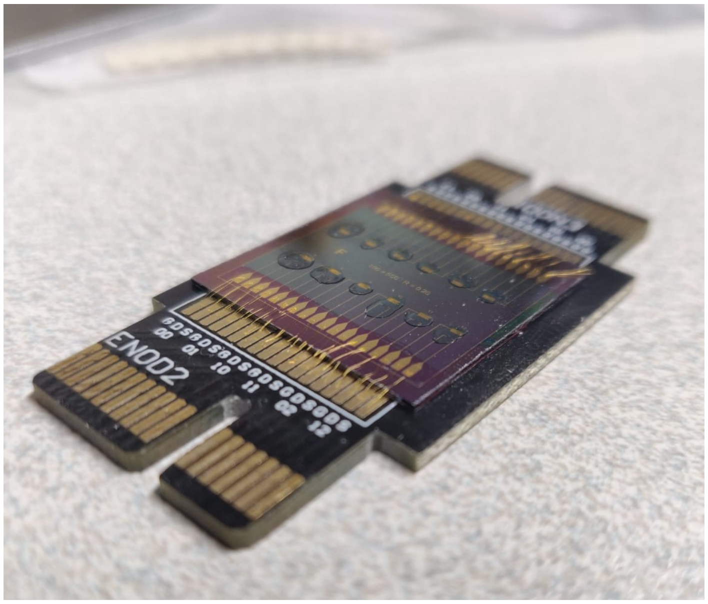

***Fig. S1**: Photo of substrate with the EC-RAM*

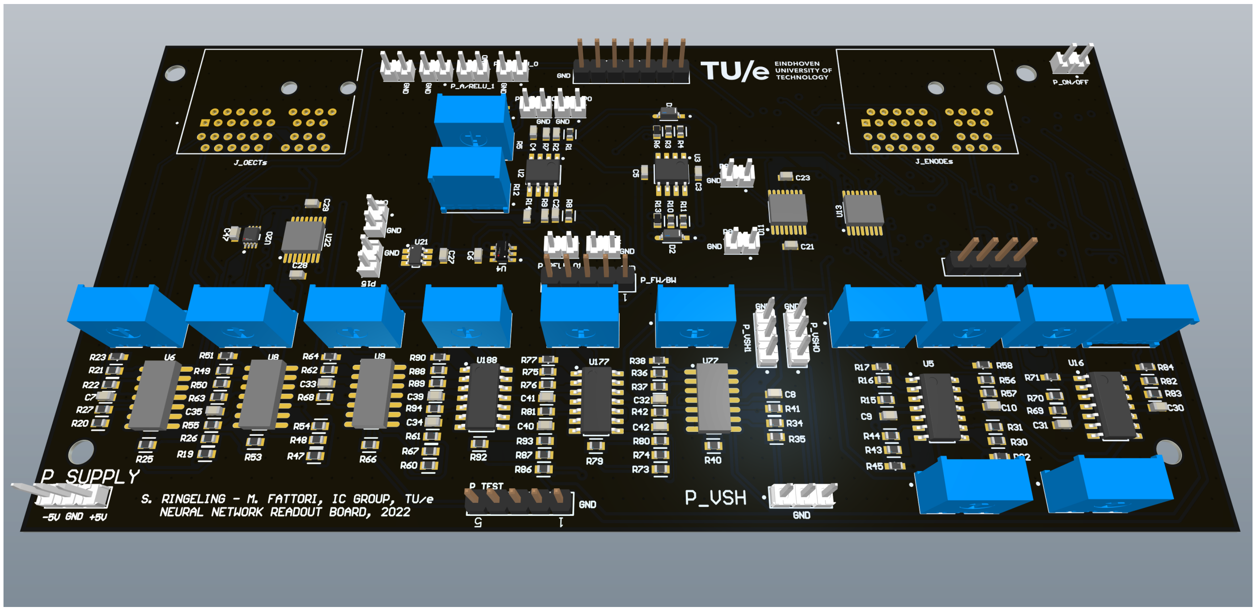

Fig. S2: Design of the PCB. *Equivalent to one network layer including the activation function (ReLU) and necessary control operations for the inference task and backpropagation algorithm.*

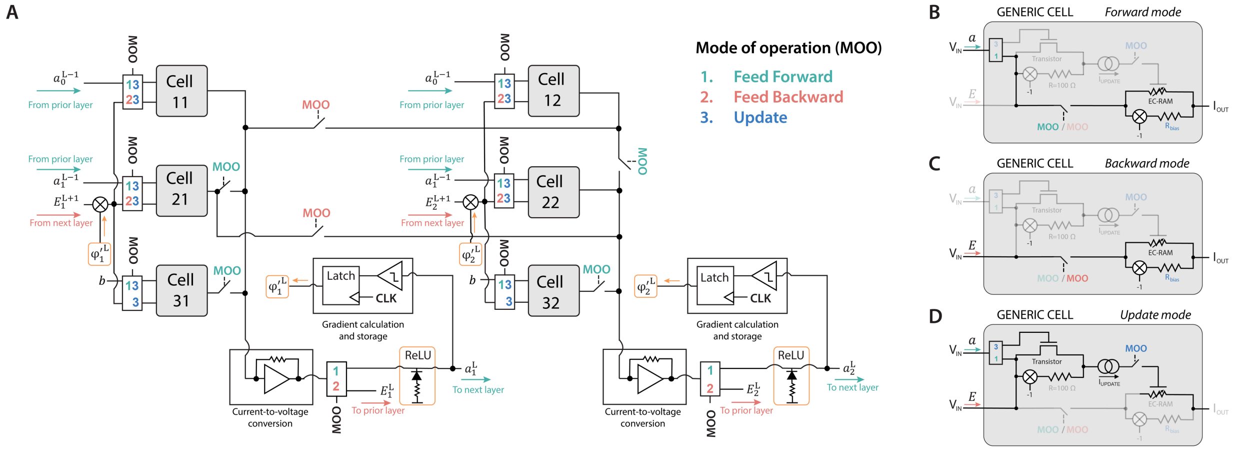

Fig. S3: *(A) Simplified block diagram of the implemented 1-layer neural network containing 6 cells. Simplified schematic of a generic cell containing an EC-RAM device and a transistor, and its operation in (B) forward propagation mode, (C) backward propagation mode and (D) update mode.*

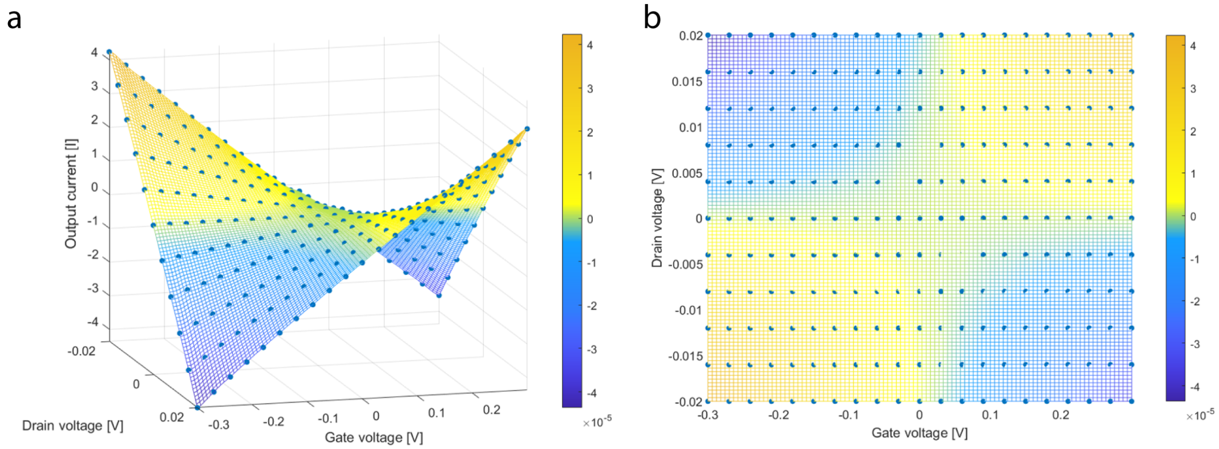

Fig. S4: Transistor multiplication measurement*. Side view (A) and top view (B) of the transistor measurement for different drain and gate voltages, using a reference resistor of 100 Ω for offsetting the conductance. The color bar represents the output current.*

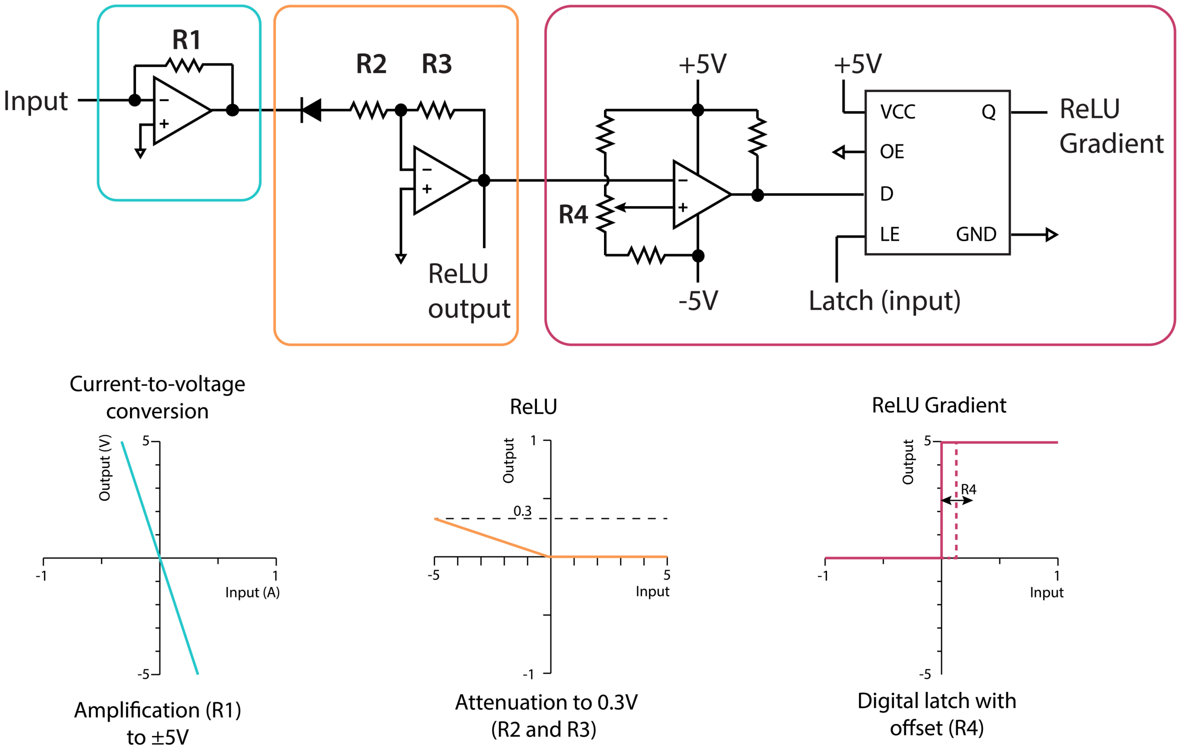

Fig. S5: Hardware ReLU*. Implementation of the rectified linear unit (ReLU) function and its gradient in hardware. The first circuit (green) converts the input (output current of the crossbar) to a voltage in the ±5V range with an amplification based on the resistor R1. Thereafter, the second part (orange) blocks all positive currents (to counteract the polarity inversion) and attenuates the negative input to a maximum 300mV output regulated with resistors R2 and R3. The last circuit (red) illustrates the storage of the ReLU gradient.*

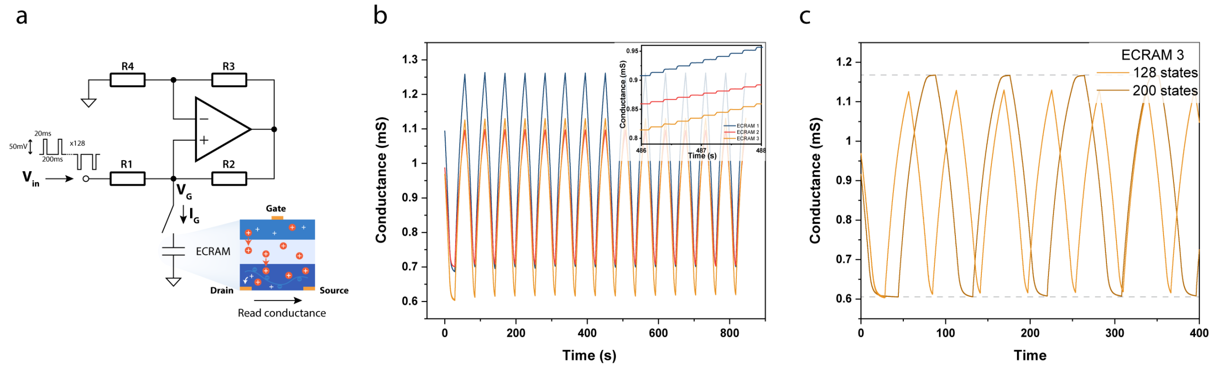

Fig. S6: Weight modulation. *(A) Schematic of the current source used to tune the conductance values of the EC-RAM. (B) Conductance modulation of 3 EC-RAM by applying 128 potentiation and depression pulses with amplitude ±50mV and duration 20ms and delay 200ms. (C) Conductance modulation of EC-RAM 3 with 128 and 200 pulses demonstrating the capping behavior of the current source. The capping mechanism is created by the ratio of R1/R2, where R1=R4 and R2=R3.*

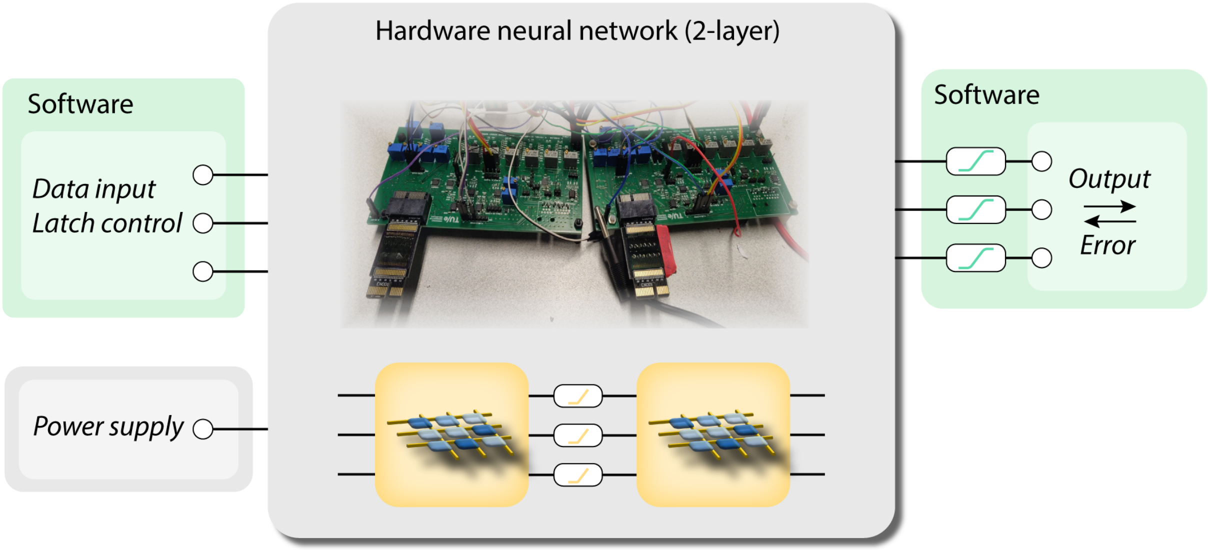

Fig. S7: Hardware neural network testing.

Table S1*: *I/O channels. *PCB to National instrument.*

Channel	PCB pins	NI I/O
1	Input crossbar (1)	AO
2	Input crossbar (2)	AO
3	Bias crossbar	AO
4	Backflowing error (1)	AO
5	Backflowing error (2)	AO
6	Mode of operation: Feedforward	DO
7	Mode of operation: Backward	DO
8	Mode of operation: Update	DO
9	Store ReLU gradient	DO
10	Apply learning	DO
11	Output crossbar (1)	AI
12	Output crossbar (2)	AI

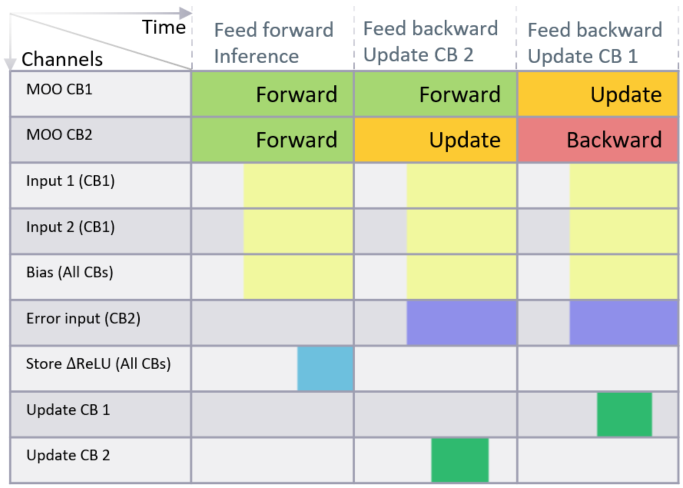

***Fig. S8**: Signal sequence generated by software to evaluate one datapoint and update the hardware layers accordingly.*

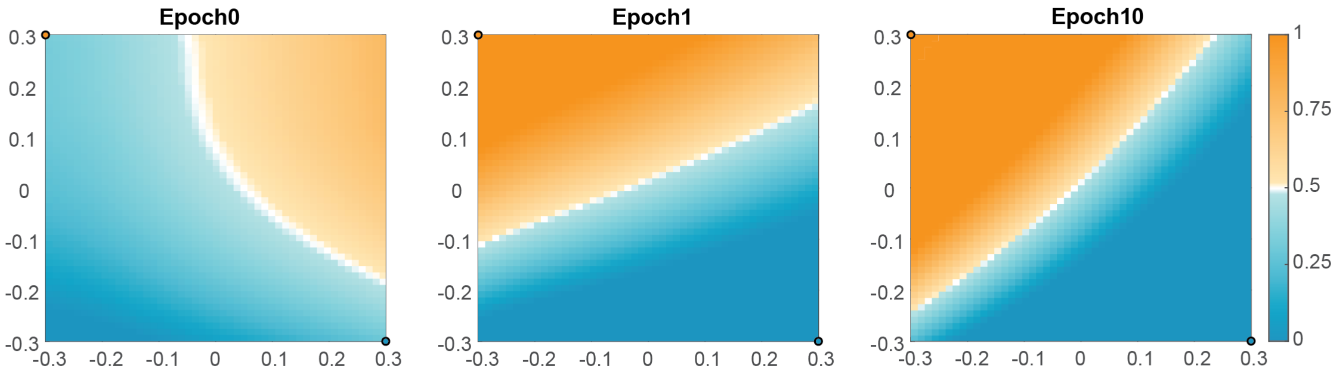

***Fig. S9**: **Decision boundaries for a single layer neural network**. Output after weight initialization (Epoch 0) and after training Epoch 1 and Epoch 10. For the evaluation of the decision boundaries the output values are recorded while sweeping over the input values from -0.3V to 0.3V in a grid of 50x50.*

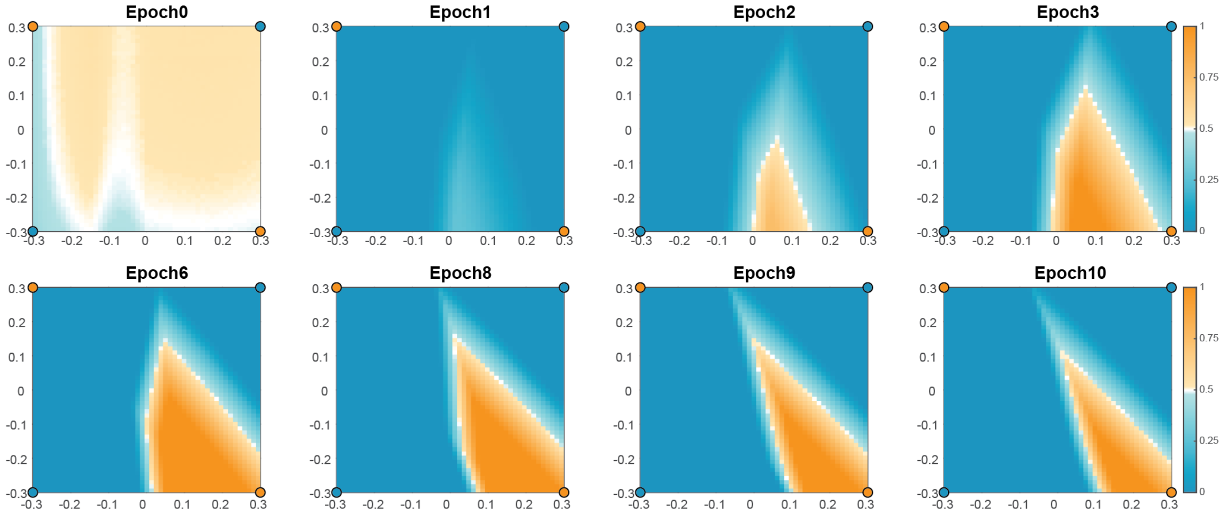

Fig. S10: Decision boundaries for a 2-layer classification after weight initialization (Epoch 0) and various other epochs. *For the evaluation of the decision boundaries the output values are recorded while sweeping over the input values from -0.3V to 0.3V in a grid of 50x50. Blue and orange data points represent class 0 and 1, respectively.*

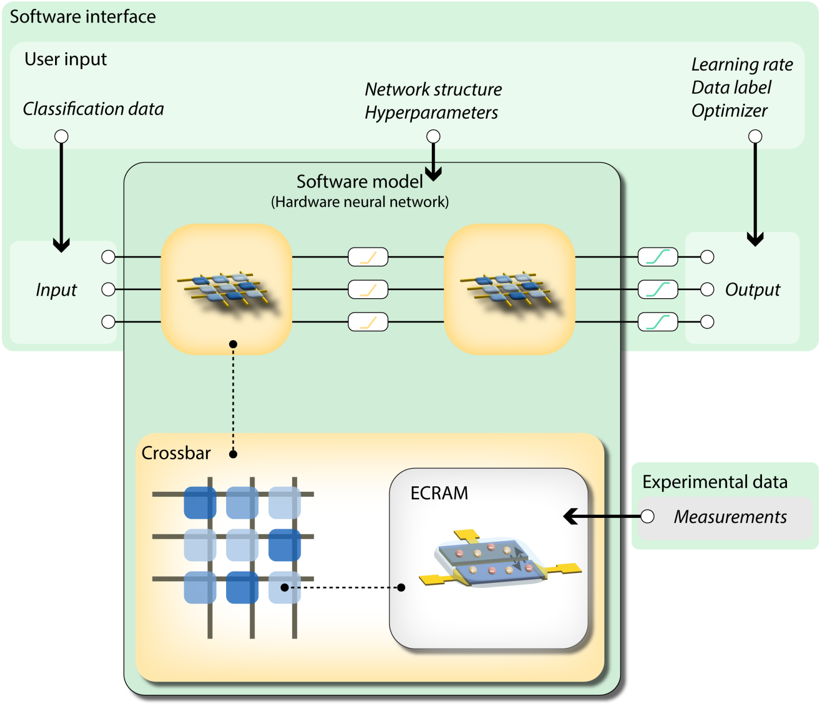

Fig. S11: *Hardware neural network model simulated with Python.*

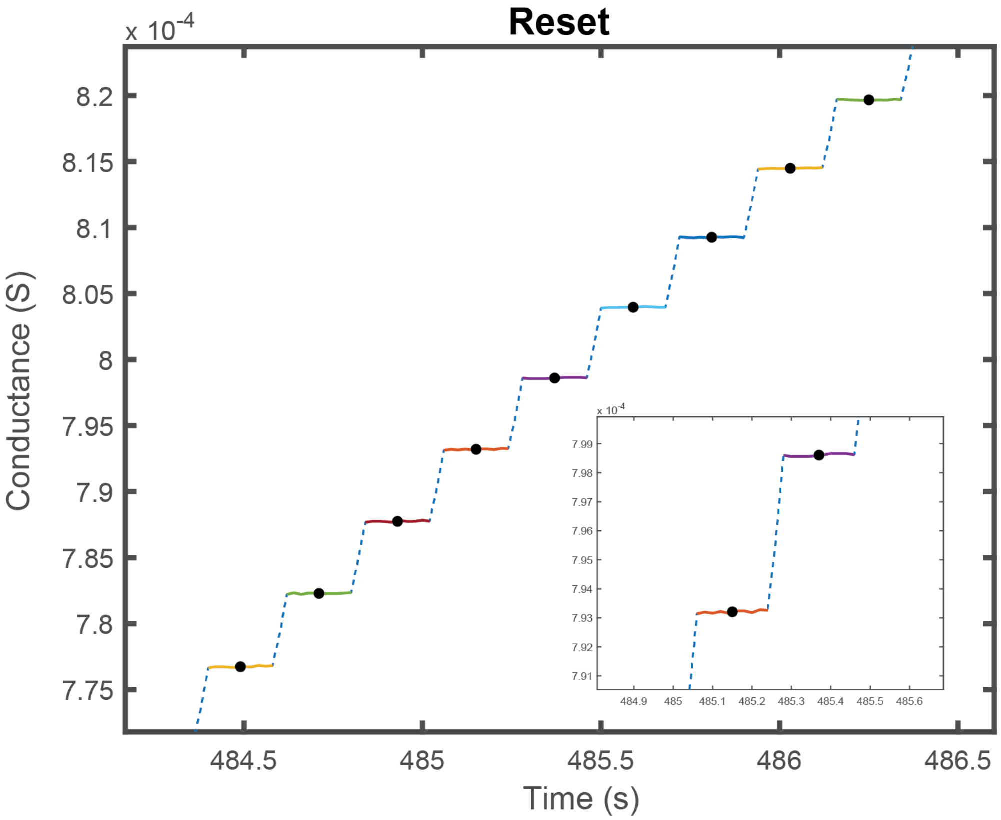

Fig. S12*: Zoom in on the conductance states of the reset pulse (-50mV, 20ms)*

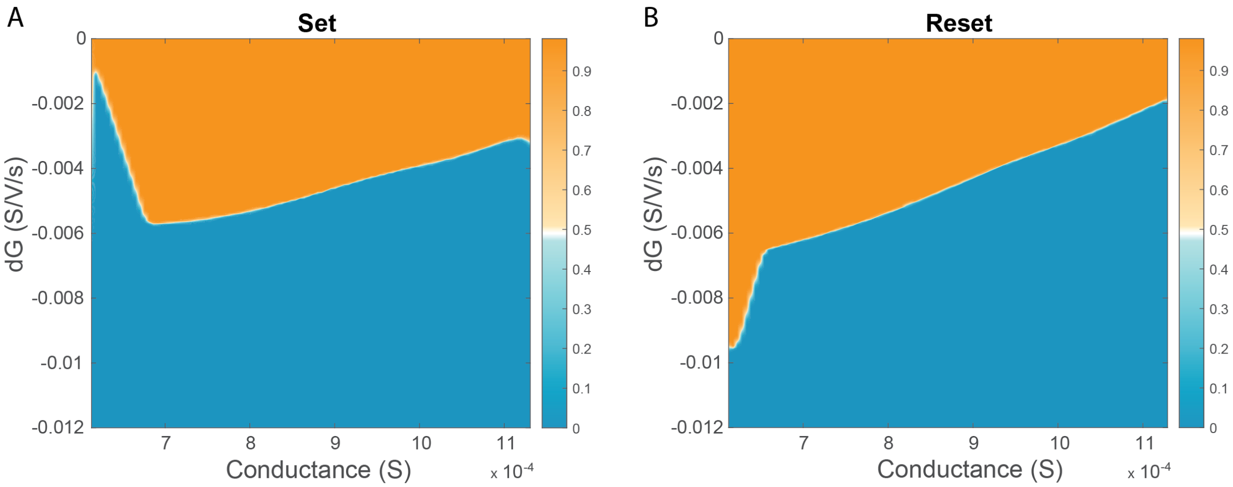

Fig. S13: *CDF plot of the conductance modulation of (A) depression and (B) potentiation.*

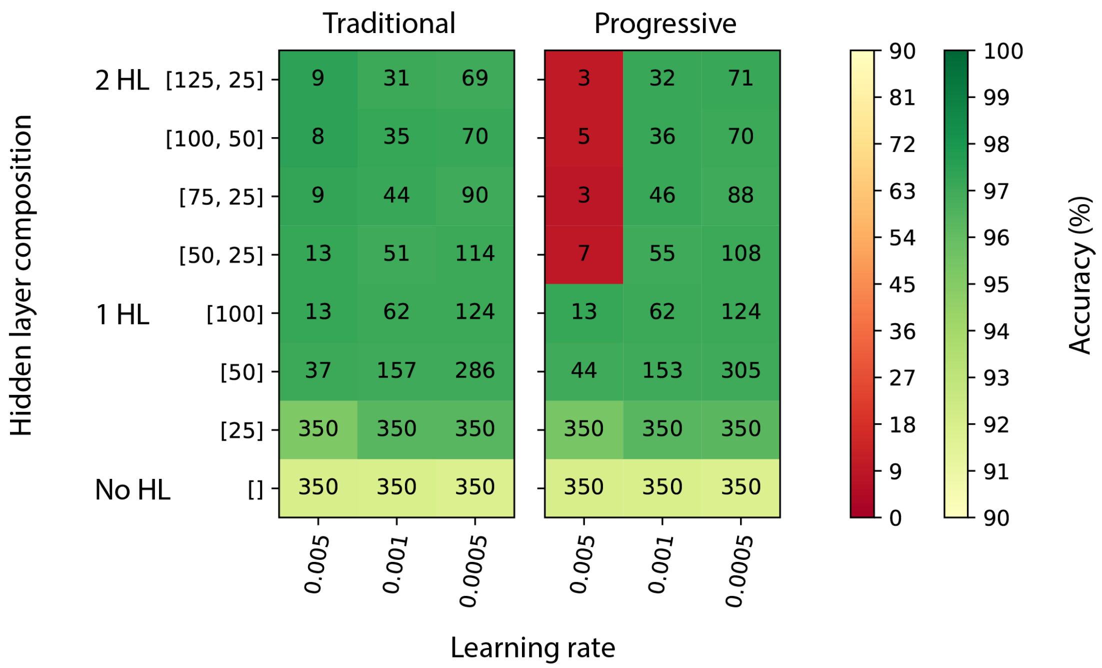

***Fig. S14**: Heatmap of the accuracy of multiple neural network architectures with varying hidden layer (HL) composition tested with different learning rates. The number of epochs required to converge to 97% accuracy is depicted in the center of the colored rectangle for each parameter.*

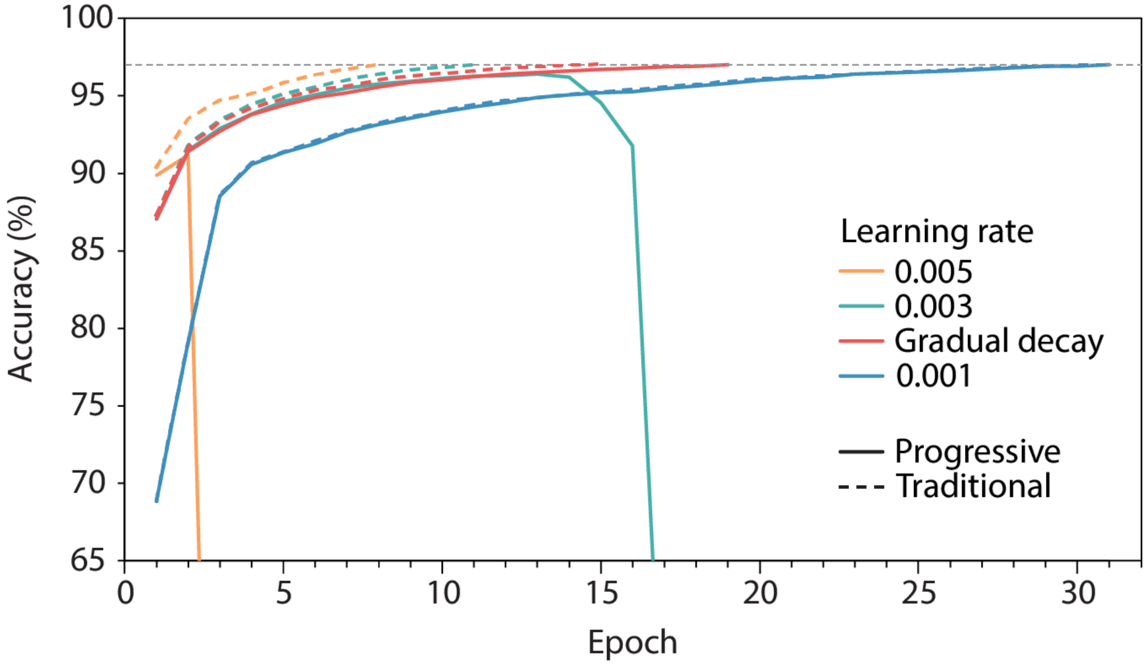

Fig. S15*: Accuracy as a function of number of epochs for various learning rates comparing the progressive (solid lines) with the traditional (dashed lines) backpropagation method. The gradual decay (red line) represents a learning rate that starts with 0.003 and reduces every epoch with 95% until a learning rate of 0.001. The gray dashed line represents the stopping criterium of 97% accuracy. The accuracy of an untrained network at epoch 0 starts at 6.28%.*

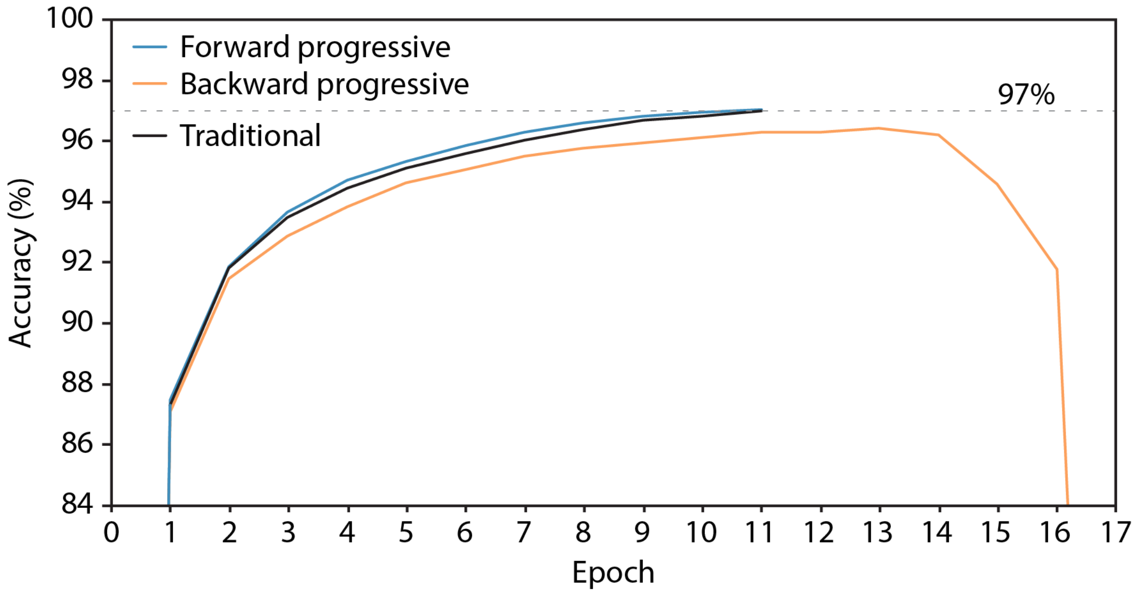

***Fig. S16**: Accuracy as a function of number of epochs comparing the forward (blue) and backward (orange) progressive with the traditional (black) backpropagation method. The neural network uses a 784×125×25×10 architecture with a learning rate of 0.003 on the MNIST digit dataset. The gray dashed line represents the stopping criterium of 97% accuracy. The accuracy of the untrained network at epoch 0 starts at 6.28%.*

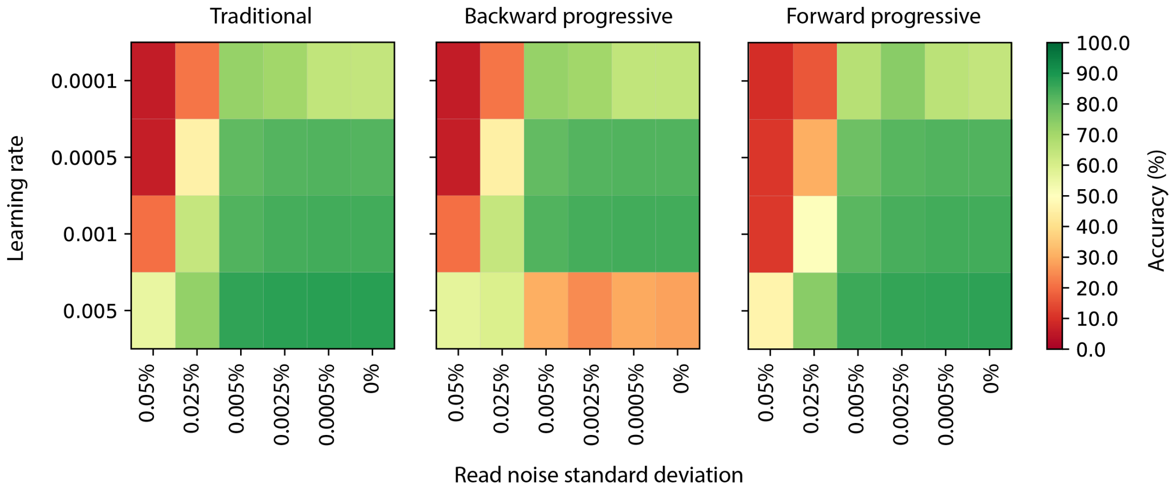

Fig. S17: *Read noise for traditional, backward progressive, and forward progressive backpropagation. Simulation executed on the MNIST Fashion dataset using 120 and 50 neurons in the two hidden layers. Accuracy after 50 epochs is indicated with color ranging from 0% (red) to 100% (green). Percentage indicates the standard deviation of the noise which value is based on the maximum range of the weight values (in this case ±0.75).*

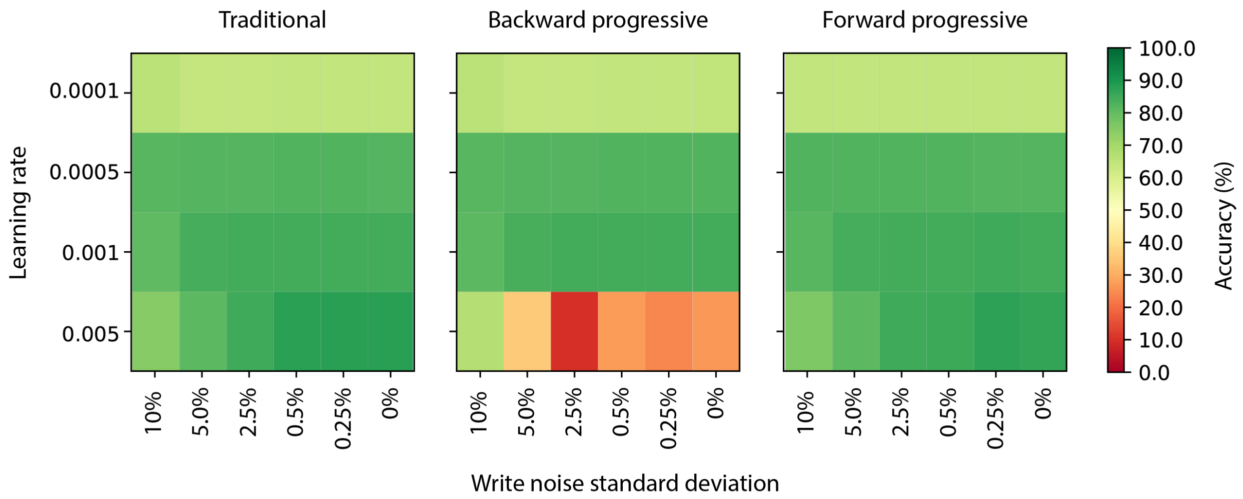

Fig. S18: *Write noise for traditional, backward progressive, and forward progressive backpropagation. Simulation executed on the MNIST Fashion dataset using 120 and 50 neurons in the two hidden layers. Accuracy after 50 epochs is indicated with color ranging from 0% (red) to 100% (green). Percentage indicates the standard deviation of the noise which value is based on the maximum range of the weight values (in this case ±0.75).*

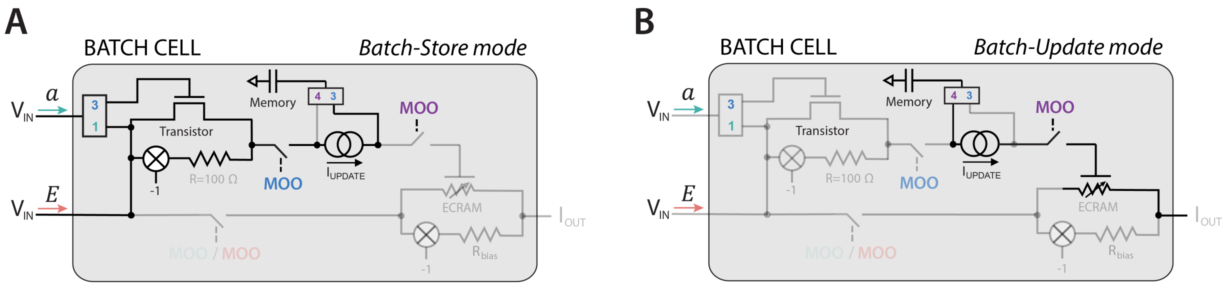

Fig. S19*: Simplified schematic of a cell for batch training containing an EC-RAM, transistor, current source, analogue memory component and switches in (A) batch-store mode and (B) batch-update mode.*

REFERENCES AND NOTES

1. D. Ielmini, H.-S. P. Wong, In-memory computing with resistive switching devices, *Nat. Electron.* **1**, 333–343 (2018).

2. M. Hu, C. E. Graves, C. Li, Y. Li, N. Ge, E. Montgomery, N. Davila, H. Jiang, R. S. Williams, J. J. Yang, Q. Xia, J. P. Strachan, Memristor-based analog computation and neural network classification with a dot product engine. *Adv. Mater.* **30**, 1705914 (2018).

3. C.-X. Xue, Y.-C. Chiu, T.-W. Liu, T.-Y. Huang, J.-S. Liu, T.-W. Chang, H.-Y. Kao, J.-H. Wang, S.-Y. Wei, C.-Y. Lee, S.-P. Huang, J.-M. Hung, S.-H. Teng, W.-C. Wei, Y.-R. Chen, T.-H. Hsu, Y.-K. Chen, Y.-C. Lo, T.-H. Wen, C.-C. Lo, R.-S. Liu, C.-C. Hsieh, K.-T. Tang, M.-S. Ho, C.-Y. Su, C.-C. Chou, Y.-D. Chih, M.-F. Chang, A CMOS-integrated compute-in-memory macro based on resistive random-access memory for AI edge devices. *Nat. Electron.* **4**, 81–90 (2021).

4. S. Ambrogio, P. Narayanan, H. Tsai, R. M. Shelby, I. Boybat, C. di Nolfo, S. Sidler, M. Giordano, M. Bodini, N. C. P. Farinha, B. Killeen, C. Cheng, Y. Jaoudi, G. W. Burr, Equivalent-accuracy accelerated neural-network training using analogue memory. *Nature* **558**, 60–67 (2018).

5. P. Yao, H. Wu, B. Gao, S. B. Eryilmaz, X. Huang, W. Zhang, Q. Zhang, N. Deng, L. Shi, H.-S. P. Wong, H. Qian, Face classification using electronic synapses. *Nat. Commun.* **8**, 15199 (2017).

6. Y. van de Burgt, E. Lubberman, E. J. Fuller, S. T. Keene, G. C. Faria, S. Agarwal, M. J. Marinella, A. Alec Talin, A. Salleo, A non-volatile organic electrochemical device as a low-voltage artificial synapse for neuromorphic computing. *Nat. Mater.* **16**, 414–418 (2017).

7. A. Melianas, T. J. Quill, G. LeCroy, Y. Tuchman, H. V. Loo, S. T. Keene, A. Giovannitti, H. R. Lee, I. P. Maria, I. McCulloch, A. Salleo, Temperature-resilient solid-state organic artificial synapses for neuromorphic computing *Sci. Adv.* **6**, eabb2958 (2020).

8. M. Onen, N. Emond, B. Wang, D. Zhang, F. M. Ross, J. Li, B. Yildiz, J. A. del Alamo, Nanosecond protonic programmable resistors for analog deep learning. *Science* **377**, 539–543 (2022).

9. J. Cui, F. An, J. Qian, Y. Wu, L. L. Sloan, S. Pidaparthy, J.-M. Zuo, Q. Cao, CMOS-compatible electrochemical synaptic transistor arrays for deep learning accelerators. *Nat. Electron.*, **6**, 292–300 (2023).

10. D. Kadetotad, Z. Xu, A. Mohanty, P.-Y. Chen, B. Lin, J. Ye, S. Vrudhula, S. Yu, Y. Cao, J. Seo, Parallel architecture with resistive crosspoint array for dictionary learning acceleration. *IEEE J. Emerg. Sel. Top. Circuits Syst.* **5**, 194–204 (2015).

11. L. Gao, I.-T. Wang, P.-Y. Chen, S. Vrudhula, J. Seo, Y. Cao, T.-H. Hou, S. Yu, Fully parallel write/read in resistive synaptic array for accelerating on-chip learning. *Nanotechnology* **26**, 455204 (2015).

12. C. Mackin, M. J. Rasch, A. Chen, J. Timcheck, R. L. Bruce, N. Li, P. Narayanan, S. Ambrogio, M. Le Gallo, S. R. Nandakumar, A. Fasoli, J. Luquin, A. Friz, A. Sebastian, H. Tsai, G. W. Burr, Optimised weight programming for analogue memory-based deep neural networks. *Nat. Commun.* **13**, 3765 (2022).

13. E. J. Fuller, S. T. Keene, A. Melianas, Z. Wang, S. Agarwal, Y. Li, Y. Tuchman, C. D. James, M. J. Marinella, J. J. Yang, A. Salleo, A. A. Talin, Parallel programming of an ionic floating-gate memory array for scalable neuromorphic computing. *Science* **364**, 570–574 (2019).

14. G. W. Burr, R. M. Shelby, S. Sidler, C. di Nolfo, J. Jang, I. Boybat, R. S. Shenoy, P. Narayanan, K. Virwani, E. U. Giacometti, B. N. Kurdi, H. Hwang, Experimental demonstration and tolerancing of a large-scale neural network (165 000 synapses) using phase-change memory as the synaptic weight element. *IEEE Trans. Electron. Devices* **62**, 3498–3507 (2015).

15. C. Li, D. Belkin, Y. Li, P. Yan, M. Hu, N. Ge, H. Jiang, E. Montgomery, P. Lin, Z. Wang, W. Song, J. P. Strachan, M. Barnell, Q. Wu, R. S. Williams, J. J. Yang, Q. Xia, Efficient and self-adaptive in-situ learning in multilayer memristor neural networks. *Nat. Commun.* **9**, 2385 (2018).

16. L. G. Wright, T. Onodera, M. M. Stein, T. Wang, D. T. Schachter, Z. Hu, P. L. McMahon, Deep physical neural networks trained with backpropagation. *Nature* **601**, 549–555 (2022).

17. M. Hermans, M. Burm, T. Van Vaerenbergh, J. Dambre, P. Bienstman, Trainable hardware for dynamical computing using error backpropagation through physical media. *Nat. Commun.* **6**, 6729 (2015).

18. P. Yao, H. Wu, B. Gao, J. Tang, Q. Zhang, W. Zhang, J. J. Yang, H. Qian, Fully hardware-implemented memristor convolutional neural network. *Nature* **577**, 641–646 (2020).

19. W. Wan, R. Kubendran, C. Schaefer, S. B. Eryilmaz, W. Zhang, D. Wu, S. Deiss, P. Raina, H. Qian, B. Gao, S. Joshi, H. Wu, H.-S. P. Wong, G. Cauwenberghs, A compute-in-memory chip based on resistive random-access memory. *Nature* **608**, 504–512 (2022).

20. G. Hinton, The forward-forward algorithm: Some preliminary investigations. arXiv:2212.13345 [cs.LG] (2022).

21. F. Alibart, E. Zamanidoost, D. B. Strukov, Pattern classification by memristive crossbar circuits using ex situ and in situ training. *Nat. Commun.* **4**, 2072 (2013).

22. M. Prezioso, F. Merrikh-Bayat, B. D. Hoskins, G. C. Adam, K. K. Likharev, D. B. Strukov, Training and operation of an integrated neuromorphic network based on metal-oxide memristors. *Nature* **521**, 61–64 (2015).

23. R. Hasan, T. M. Taha, Enabling back propagation training of memristor crossbar neuromorphic processors, in *2014 International Joint Conference on Neural Networks (IJCNN)* (IEEE, 2014), pp. 21–28.

24. S. Lim, J.-H. Bae, J.-H. Eum, S. Lee, C.-H. Kim, D. Kwon, B.-G. Park, J.-H. Lee, Adaptive learning rule for hardware-based deep neural networks using electronic synapse devices. *Neural Comput. Appl.* **31**, 8101-8116 (2019).

25. R. Karakiewicz, R. Genov, G. Cauwenberghs, 1.1 TMACS/mW fine-grained stochastic resonant charge-recycling array processor. *IEEE Sens. J.* **12**, 785–792 (2012).

26. Z. Cheng, D. Soudry, Z. Mao, Z. Lan, Training binary multilayer neural networks for image classification using expectation backpropagation. arXiv:1503.03562 [cs.NE] (2015).

27. D. Soudry, D. D. Castro, A. Gal, A. Kolodny, S. Kvatinsky, Memristor-based multilayer neural networks with online gradient descent training. *IEEE Trans. Neural Netw. Learn. Syst.* **26**, 2408–2421 (2015).

28. A. Sebastian, M. Le Gallo, R. Khaddam-Aljameh, E. Eleftheriou, Memory devices and applications for in-memory computing. *Nat. Nanotechnol.* **15**, 529–544 (2020).

29. S. Agarwal, S. J. Plimpton, D. R. Hughart, A. H. Hsia, I. Richter, J. A. Cox, C. D. James, M. J. Marinella, Resistive memory device requirements for a neural algorithm accelerator, in *2016 International Joint Conference on Neural Networks (IJCNN)* (IEEE, 2016), pp. 929–938.

30. A. A. Talin, Y. Li, D. A. Robinson, E. J. Fuller, S. Kumar, ECRAM materials, devices, circuits and architectures: A perspective. *Adv. Mater.* **35**, 2204771 (2023).

31. Y. LeCun, C. Cortes, C. J. C. Burges, The MNIST Database of Handwritten Digits (1998); http://yann.lecun.com/exdb/mnist/.

32. L. S. Hu, An energy-efficient solid-state organic device array for neuromorphic computing. *IEEE Trans. Electron. Devices* **70**, 6520–6525 (2023).

33. B. Moons, K. Goetschalckx, N. Van Berckelaer, M. Verhelst, Minimum energy quantized neural networks. arXiv:1711.00215 [cs.NE] (2017).

34. E. García-Martín, C. F. Rodrigues, G. Riley, H. Grahn, Estimation of energy consumption in machine learning. *J. Parallel Distrib. Comput.* **134**, 75–88 (2019).

35. S. Han, J. Pool, J. Tran, W. J. Dally, Learning both weights and connections for efficient neural networks. arXiv:1506.02626 [cs.NE] (2015).

36. B. D. Hoskins, M. W. Daniels, S. Huang, A. Madhavan, G. C. Adam, N. Zhitenev, J. J. McClelland, M. D. Stiles, Streaming batch eigenupdates for hardware neural networks. *Front. Neurosci.* **13**, 793 (2019).
